# Dynamic behaviors of a modified SIR model in epidemic diseases using nonlinear incidence and recovery rates

**DOI:** 10.1371/journal.pone.0175789

**Published:** 2017-04-20

**Authors:** Gui-Hua Li, Yong-Xin Zhang

**Affiliations:** Department of Mathematics, North University of China, Taiyuan, Shan’xi 030051, P. R. China; Shanxi University, CHINA

## Abstract

The transmission of infectious diseases has been studied by mathematical methods since 1760s, among which SIR model shows its advantage in its epidemiological description of spread mechanisms. Here we established a modified SIR model with nonlinear incidence and recovery rates, to understand the influence by any government intervention and hospitalization condition variation in the spread of diseases. By analyzing the existence and stability of the equilibria, we found that the basic reproduction number $R_{0}$ is not a threshold parameter, and our model undergoes backward bifurcation when there is limited number of hospital beds. When the saturated coefficient *a* is set to zero, it is discovered that the model undergoes the Saddle-Node bifurcation, Hopf bifurcation, and Bogdanov-Takens bifurcation of codimension 2. The bifurcation diagram can further be drawn near the cusp type of the Bogdanov-Takens bifurcation of codimension 3 by numerical simulation. We also found a critical value of the hospital beds *b*_*c*_ at $R_{0}<1$ and sufficiently small *a*, which suggests that the disease can be eliminated at the hospitals where the number of beds is larger than *b*_*c*_. The same dynamic behaviors exist even when *a* ≠ 0. Therefore, it can be concluded that a sufficient number of the beds is critical to control the epidemic.

## Introduction

Since the development of the first dynamic model of smallpox by Bernoulli in 1760, various mathematical models have been employed to study infectious diseases [1] in order to reveal the underlying spread mechanisms that influence the transmission and control of these diseases. Among them, Kermack and Mckendrick [2] initiated a famous SIR type of compartmental model in 1927 for the plague studies in Mumbai, and succeed in unveiling its epidemiological transition. Since then, mathematical modeling has become an important tool to study the transmission and spread of epidemic diseases.

In the modeling of infectious diseases, the incidence function is one of the important factors to decide the dynamics of epidemic models. Bilinear and standard incidence rates, both monotonically increasing functions of the total of infected class, have been frequently used in early epidemic models [3]. In those models, the dynamics of models are relatively simple and almost determined by the basic reproduction number $R_{0}$: the disease will be eliminated if $R_{0}<1$, otherwise, the disease will persist. However, intervention strategies, such as isolation, quarantine, mask-wearing and medical report about emerging infectious diseases, play an vital role in controlling the spread, sometimes contributing to the eradication of diseases. For instance, the SARS in 2003 and novel influenza pandemic in 2009 have been well controlled by taking these intervention actions [4–15]. Hence, it is essential to expand the modeling studies to the investigation of the combined effects of these major intervention strategies. The generalized models will provide further understanding of the transmission mechanisms, and modify guidelines for public health in control of the spread of infectious diseases.

In recent years, a number of compartmental models have been formulated to explore the impact of intervention strategies on the transmission dynamics of infectious diseases. If denote the total number of hospital individuals, exposed and infectious as *N*, *E* and *I* respectively, Liu et al [16], Cui et al [17, 18] used *βe*^−*mI*^, $\beta e^{-\alpha_{1}E-\alpha_{2}I-\alpha_{3}H}$ and *c*_1_ − *c*_2_ *f*(*I*) to study the impact of media coverage on the dynamics of epidemic models, respectively. However, people may adjust their behaviors according to these government intervention. Therefore, Ruan and Xiao [19] set incidence function in the form of $f_{1}\left(I\right)S=\frac{KIS}{1+\alpha I^{2}}$(a special case of $\frac{kI^{p}S}{1+aI^{q}}$ [20, 21]) to include the above “psychological” effect: when the number of infectious individuals increases and is reported through social media, the susceptible individuals will stay alert spontaneously to reduce any unnecessary contact with others, thus lowering the contact and transmission incidence.

On the other hand, medical treatments, determining how well the diseases are controlled, are normally expressed as constant recovery rates in the current models. These recovery rates depend on various health systems and hospitalization conditions, such as the capacity of the hospitals and effectiveness of the treatments. Advanced models (see [22–24]) started to corporate the limited medical resources into the spread dynamics of infectious diseases. In the literature [22], Wang and Ruan first introduced a piece-wise treatment function in an SIR model,

$$\begin{array}{c} T\left(I\right)=\left\{\begin{array}{cc} r & I>0,\\ 0 & I=0. \end{array}\right. \end{array}$$

where the maximal treatment capacity was used to cure infectives so that the epidemic of disease can be controlled. This situation occurs only if the infectious disease needs to be eliminated due to its threats to public. They discovered that the model undergoes Saddle-Node bifurcation, Hopf bifurcation and Bogdanov-Takens bifurcation, standing for the collision of two equilibria, the existance of periodic diseases, and two varying parameters in system, respectively. Wang [23] further modified the treatment rate to be proportinal to the number of infectives before the capacity of hospital was reached, by

$$\begin{array}{c} T\left(I\right)=\left\{\begin{array}{cc} rI & 0\leq I\leq I_{0},\\ rI_{0} & I>I_{0}. \end{array}\right. \end{array}$$ (1)

The model was then found to perform backward bifurcation [23], indicating that the basic reproduction number was no longer a threshold.

In common hospital settings, the number of beds is an indicator of health resources, particularly the medical treatments of the infectives. Under this consideration, Shan and Zhu [24] defined the recovery rate as a function of *b*, the number of hospital beds, and *I*, the number of infectives.

$$\begin{array}{c} \mu =\mu \left(b,I\right)=\mu_{0}+\left(\mu_{1}-\mu_{0}\right)\frac{b}{I+b} \end{array}$$ (2)

where *μ*_0_ is the minimum per capita recovery rate, and *μ*_1_ the maximum per capita recovery rate. They chose the standard incidence rate and discovered the complicated dynamics including Saddle-Node bifurcation, Backward bifurcation and Bogdanov-Takens bifurcation of codimension 3, which means that the recovery rate contributes to the rich dynamics of epidemic models.

**Fig 1:**
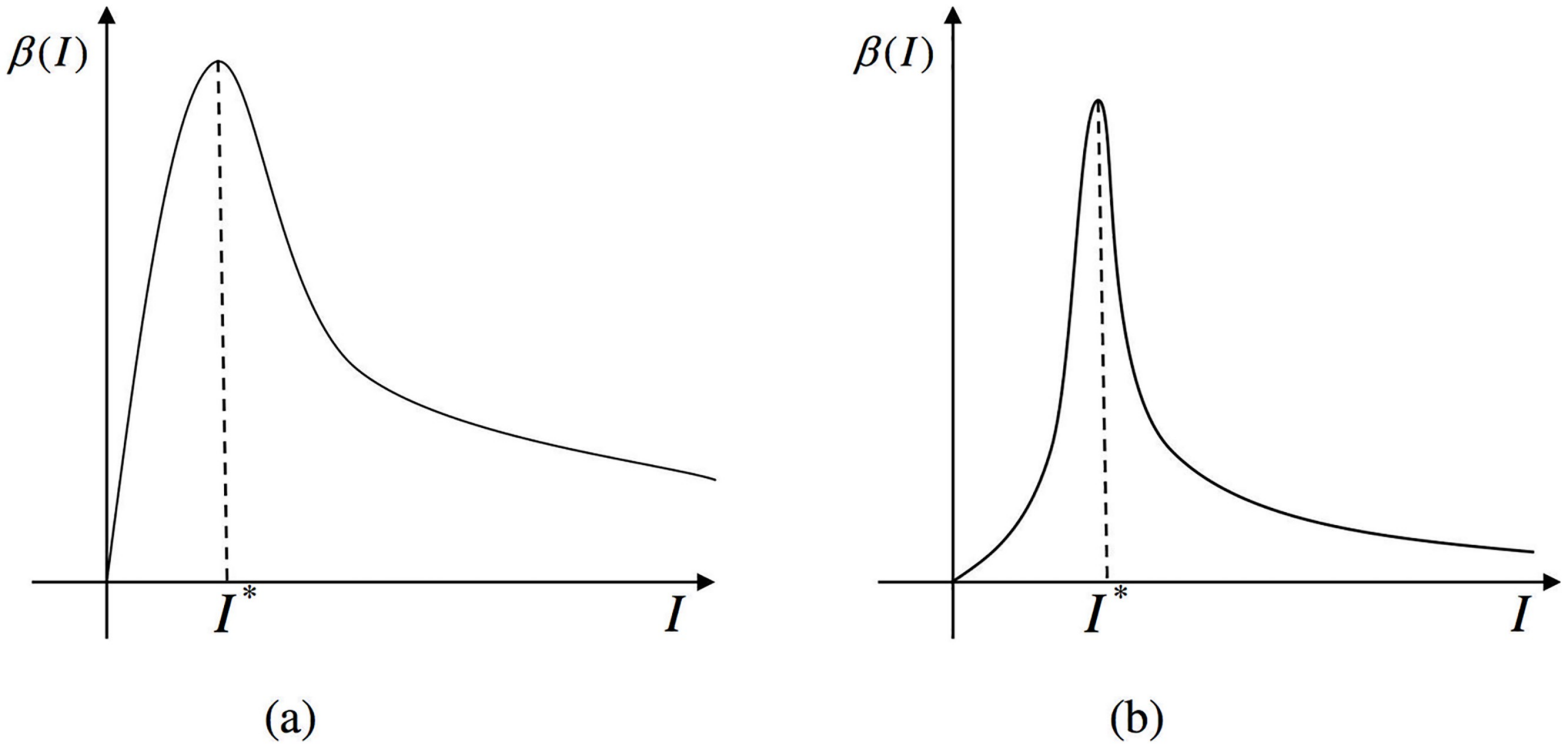
Graphs of incidence rate function. (a) *c* ≥ 0; (b) $-2\sqrt{a}<c<0$.

Our strategy thus becomes, both government intervention and hospitalization condition need to be incorporated to achieve a better control of the emerging infectious. Therefore, the incidence rate is expressed as

$$\begin{array}{c} \beta \left(I\right)=\frac{\beta I}{aI^{2}+cI+1}, \end{array}$$

where *a* is positive constant and $c>-2\sqrt{a}$ (so that *aI*^2^ + *cI* + 1 > 0 for all *I* > 0 and hence *β*(*I*) > 0 for all *I* > 0). When the threshold of the number of infected individuals I* is reached, the contact transmission rate starts to decrease as the number of infected individuals grows. As shown in Fig 1, the incidence *β*(*I*) increases to its maximum and then decreases to zero as *I* tends to infinity, which explains the phenomenon where the rate of contacting between infected *I* and susceptible *S* decreases after government intervention. We use the same expression of hospitalization conditions as the literature [24], and the following model is then established,

$$\begin{array}{c} \left\{\begin{array}{ccc} \frac{dS}{dt} & = & A-dS-\frac{\beta SI}{1+cI+aI^{2}},\\ \frac{dI}{dt} & = & \frac{\beta SI}{1+cI+aI^{2}}-dI-\alpha I-\mu \left(b,I\right)I,\\ \frac{dR}{dt} & = & \mu (b,I)I-dR, \end{array}\right. \end{array}$$ (3)

where *A* is the recruitment rate of the susceptible population, *d* the natural death of the population, and *α* the per capita disease-induced death rate, respectively.

For system (3), the cone *R*^3+^ is a positive invariant. The *C*^1^ smoothness of the right side of system (3) implies the local existence and uniqueness of the solution with initial values in *R*^3+^. Adding up the three equations of system (3), we get *N*′(*t*) = *A* − *dN* − *αI*. Therefore, all solutions in the first octant approach, entering or staying inside the set, are defined by

$$\begin{array}{c} D=\{\left(S,I,R\right)|S\geq 0,I\geq 0,R\geq 0,S+I+R\leq \frac{A}{d}\}. \end{array}$$

This paper will be organized as follows. In section 2, we study the existence of the equilibria of our model. In section 3, we study the stability of the equilibria. In section 4, we examine the dynamics of the model by first looking at the backward bifurcation of system, then the much complicated Hopf bifurcation and Bogdanov-Takens bifurcation of codimension 2 and 3. We summarize our results and discuss the epidemiological significance of the number of hospital beds and intervention strategies in section 5.

## Existence of equilibria

For simplicity we will focus on the case when *c* = 0. If *c* ≠ 0, but *c* is in small neighborhood of zero, the behaviors of model still exist. Our model thus becomes,

$$\begin{array}{c} \left\{\begin{array}{ccc} \frac{dS}{dt} & = & A-dS-\frac{\beta SI}{1+aI^{2}},\\ \frac{dI}{dt} & = & \frac{\beta SI}{1+aI^{2}}-dI-\alpha I-\left(\mu_{0}+\left(\mu_{1}-\mu_{0}\right)\frac{b}{b+I}\right)I,\\ \frac{dR}{dt} & = & (\mu_{0}+\left(\mu_{1}-\mu_{0}\right)\frac{b}{b+I})I-dR. \end{array}\right. \end{array}$$ (4)

Since the first two equations are independent of the third, it suffices to consider the first two equations. Thus, we will focus on the reduced model in the following discussions.

$$\begin{array}{c} \left\{\begin{array}{ccc} \frac{dS}{dt} & = & A-dS-\beta \frac{SI}{1+aI^{2}}\\ \frac{dI}{dt} & = & \beta \frac{SI}{1+aI^{2}}-dI-\alpha I-\left(\mu_{0}+\left(\mu_{1}-\mu_{0}\right)\frac{b}{b+I}\right)I. \end{array}\right. \end{array}$$ (5)

We find equilibria by setting the right hand of system (5) equal to zero:

$$\begin{array}{c} \left\{\begin{array}{cc} & A-dS-\beta \frac{SI}{1+aI^{2}}=0,\\ & \beta \frac{SI}{1+aI^{2}}-dI-\alpha I-\mu \left(b,I\right)I=0. \end{array}\right. \end{array}$$ (6)

Obviously, a trivial solution of Eq (6) is $E_{0}\left(S,I\right)=\left(\frac{A}{d},0\right)$, a disease free equilibrium(DFE). For *E*_0_, by using the formula in [25], one can calculate the reproduction number

$$\begin{array}{c} R_{0}=\frac{\beta A}{d(\alpha +d+\mu_{1})}. \end{array}$$ (7)

For any positive equilibrium *E*(*S*, *I*), also called endemic equilibrium, when exists, its *S* and *I* coordinates satisfy

$$\begin{array}{c} \;S\left(I\right)=\frac{A-(d+\alpha +\mu (b,I))I}{d}, \end{array}$$ (8)

where the *I* coordinate will be the positive root of the following cubic equation

$$\begin{array}{c} \;f\left(I\right)=AI^{3}+BI^{2}+CI+D=0, \end{array}$$ (9)

where

$$\begin{array}{ll} A=ad\delta_{0}, & B=bad\delta_{1}+\beta \delta_{0},\\ C=\beta b\delta_{1}+d\delta_{0}-\beta A, & D=bd\delta_{1}\left(1-{\mathbb{R}}_{0}\right),\\ \delta_{i}=d+\alpha +\mu_{i},\;i=0,1. & \end{array}$$

Let

$$\begin{array}{c} A_{1}=B^{2}-3AC,B=BC-9AD,C=C^{2}-3BD \end{array}$$

Denote Δ_0_ the discriminant of *f*(*I*) with respect to *I*, then

$$\begin{array}{c} \Delta_{0}=B^{2}-4A_{1}C. \end{array}$$

**Fig 2:**
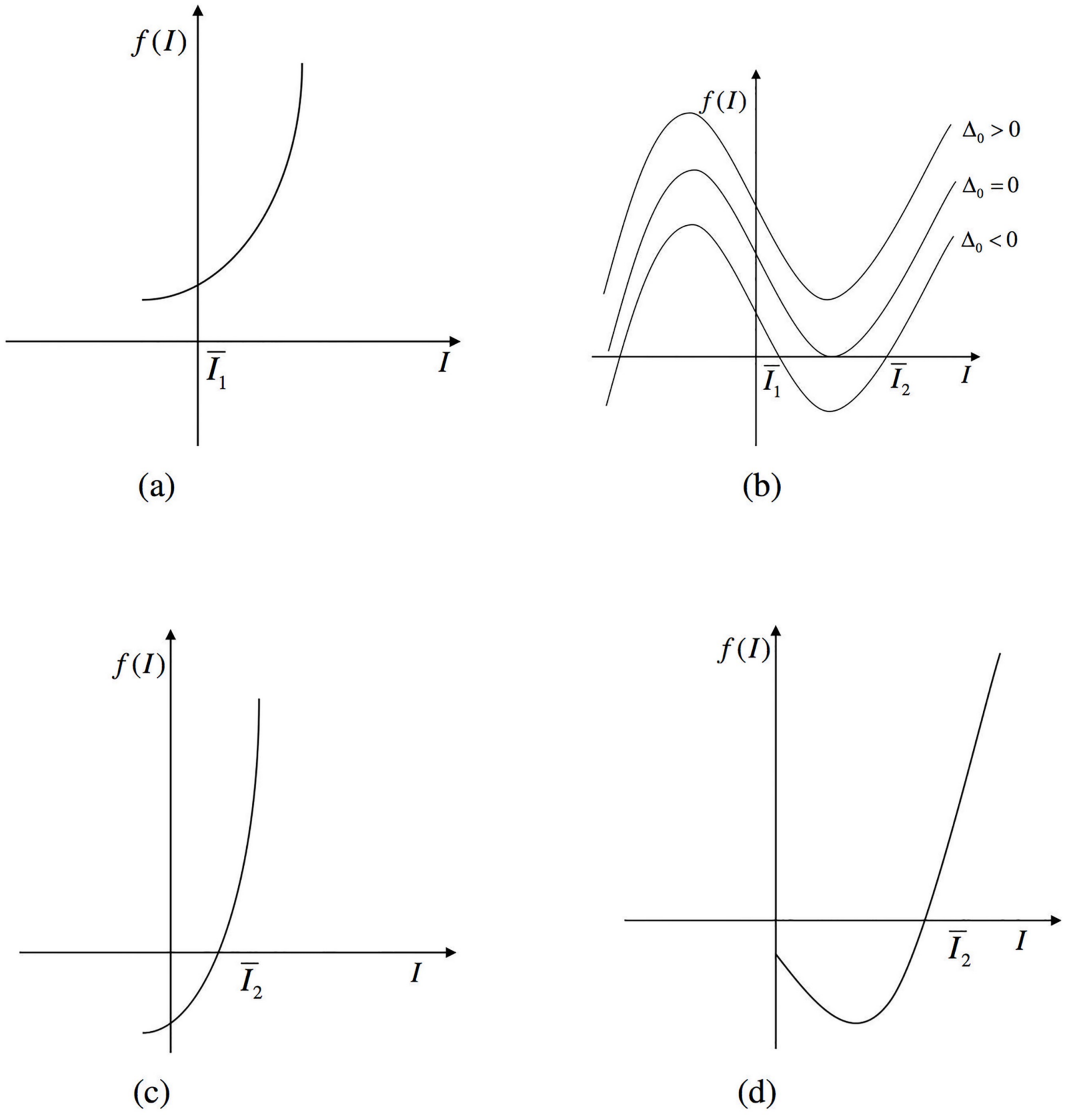
The positive roots of *f*(*I*). (a) $R_{0}<1,C>0$; (b) $R_{0}<1,C<0$; (c) $R_{0}>1,C>0$; (d) $R_{0}>1,C<0$.

Note that $f\left(0\right)=bd\delta_{1}\left(1-R_{0}\right)$ and f′(0)=C. As shown in Fig 2, we have the following cases about the positive roots of *f*(*I*):

Case 1: $R_{0}>1$. In this case, D<0. It is found that there is a unique positive root of *f*(*I*) = 0, regardless of the sign of *C* from Fig 1(c) and 1(d).
Case 2: $R_{0}<1$. In this case, D>0. If C>0, equation *f*(*I*) = 0 has no positive solution (see Fig 1(a)). If C<0, similar to Lemma 2.1 described by Huang and Ruan [26], the following conclusions can be drawn as shown by Fig 1(b).
  (a) Δ_0_ < 0, there are two positive solutions of the equation.
  (b) Δ_0_ = 0, there is unique positive solution of the equation.
  (c) Δ_0_ > 0, we find that Eq (9) has no positive solution.
Case 3: $R_{0}=1$, Eq (9) becomes
  $$\begin{array}{c} \;f\left(I\right)=AI^{3}+BI^{2}+CI=0 \end{array}$$ (10)
  If C<0, Eq (9) has a unique positive root. If C>0, Eq (9) has no positive root. Note that C>0 means $b>\frac{d(\mu_{1}-\mu_{0})}{\beta \delta_{1}}$. Thus, we get the following theorem about the equilibrium of the model.

**Theorem 0.1**. *For* system (5) *with positive parameters*,

(1) *the disease-free equilibrium E*_0_ *always exists*,
(2) *when* $R_{0}>1$, *system has a unique endemic equilibrium*,
(3) *when* $R_{0}<1$, *and*
  (a) C>0, system (5) *does not have endemic equilibrium*.
  (b) C<0, Δ_0_ < 0, *there exist two endemic equilibria* $E_{1}\left({\bar{I}}_{1},{\bar{S}}_{1}\right)$ *and* $E_{2}\left({\bar{I}}_{2},{\bar{S}}_{2}\right)$, *and when* Δ_0_ = 0 these two endemic equilibria coalesce into the same endemic equilibrium E*; otherwise system (5) *has no endemic equilibrium*.
(4) *when* $R_{0}=1$, *there exists a unique endemic equilibrium if and only if* C<0 *i*.*e*. $b<\frac{d(\mu_{1}-\mu_{0})}{\beta \delta_{1}}$; *otherwise there is no endemic equilibrium*.

**Remark 0.2**. *By calculation*, *we get that*

$$\begin{array}{c} \begin{array}{ccc} \Delta_{0}\left(a\right) & = & -3\beta^{2}\delta_{0}\Delta +O\left(a\right),\\ \Delta & = & \beta^{2}\delta_{1}^{2}b^{2}+2\beta \left(2\beta A\delta_{0}-d\delta_{0}\delta_{1}-\beta A\delta_{1}\right)b+{\left(d\delta_{0}-\beta A\right)}^{2}. \end{array} \end{array}$$ (11)

*When a is sufficiently small and tends to zero*, *the sign of* Δ_0_ *will be determined by the zero power of a*. *Therefore*, Δ_0_(*a*) → Δ_0_(0) = −3*β*^2^*δ*_0_Δ *as a* → 0. *In addition*, Δ = 0 *if and only if* $R_{0}=R_{0}^{c}$. *Here*

$$\begin{array}{c} R_{0}^{c}=1-\frac{C^{2}}{4Bbd(d+\alpha +\mu_{1})}. \end{array}$$

## Stability analysis of equilibria

In order to discuss the stability of equilibrium, we need the Jacobian matrix of system (5) at any equilibrium *E*(*S*, *I*). If we denote the Jacobian as *J*(*E*) = (*j*_*ij*_)_2×2_, *i*, *j* = 1, 2, then a straightforward calculation gives

$$\begin{array}{c} \begin{array}{ccc} & j_{11}=-d-\frac{\beta I}{1+aI^{2}}, & j_{12}=-\frac{\beta S}{1+aI^{2}}+\frac{2a\beta SI^{2}}{{\left(1+aI^{2}\right)}^{2}},\\ & j_{21}=\frac{\beta I}{1+aI^{2}}, & j_{22}=-\delta_{0}+\frac{\beta S}{1+aI^{2}}-\frac{2a\beta SI^{2}}{{\left(1+aI^{2}\right)}^{2}}+\frac{(\mu_{1}-\mu_{0})bI}{{\left(b+I\right)}^{2}}-\frac{(\mu_{1}-\mu_{0})b}{b+I}. \end{array} \end{array}$$ (12)

Firstly, we present a theorem about the disease-free equilibrium *E*_0_(*A*/*d*, 0).

**Theorem 0.3**. *E*_0_ *is an attracting node if* $R_{0}<1$, *and hyperbolic saddle if* $R_{0}>1$. *When* $R_{0}=1$, *E*_0_ *is a saddle-node of codimension* 1 *if* $b\neq \frac{d^{2}\left(\mu_{1}-\mu_{0}\right)}{\beta^{2}A}$ *and attracting semi-hyperbolic node of codimension* 2 *if* $b=\frac{d^{2}\left(\mu_{1}-\mu_{0}\right)}{\beta^{2}A}$.

*Proof*. For system (5), −*d* and $\delta_{1}\left(R_{0}-1\right)$ are two eigenvalues of *J*(*E*_0_). So, *E*_0_ is an attracting node if $R_{0}<1$, and unstable if $R_{0}>1$. When $R_{0}=1$, the second eigenvalue becomes zero. In order to analyze the behavior of *E*_0_, we linearize system (5) and use the transformation of $X=S+\frac{\beta A}{d^{2}}I$, *Y* = *I*,

$$\{\begin{array}{l} \frac{dX}{dt}=-dX+P\left(X,Y\right),\\ \frac{dY}{dt}=-\left(\frac{\mu_{0}-\mu_{1}}{b}+\frac{\beta^{2}A}{d^{2}}\right)Y^{2}+Q\left(X,Y\right), \end{array}$$ (13)

where *P*(*X*, *Y*) and *Q*(*X*, *Y*) represent the higher order terms. Obviously, *E*_0_ is a saddle-node if $b\neq \frac{d^{2}\left(\mu_{1}-\mu_{0}\right)}{\beta^{2}A}$. Otherwise, i.e., $b=\frac{d^{2}\left(\mu_{1}-\mu_{0}\right)}{\beta^{2}A}$, applying the center manifold theorem, system (5) becomes

$$\{\begin{array}{l} \frac{dX}{dt}=-dX+P\left(X,Y\right),\\ \frac{dY}{dt}=-\left(\frac{\beta Aa}{d}+\frac{\beta^{3}A\delta_{0}}{d^{3}}\right)Y^{3}+Q_{1}\left(X,Y\right), \end{array}$$ (14)

where *Q*_1_(*X*, *Y*) represents the higher order term. Thus, *E*_0_ is an attracting semi-hyperbolic node of codimension 2.

**Theorem 0.4**. *If* *dδ*_0_ > *βA*, *E*_0_ *is globally asymptotically stable*.

*Proof*. If *dδ*_0_ > *βA*, it is obvious that $R_{0}<1$ and C>0. From Theorem 0.1 and 0.3, *E*_0_ is the unique attracting node of system (5). In order to prove that the disease free equilibrium *E*_0_ is globally and asymptotically stable, we construct the following Liapunov function:

$$\begin{array}{c} V\left(S,I\right)=\frac{A}{d}\left(\frac{dS}{A}-\ln\frac{dS}{A}\right)+I. \end{array}$$ (15)

It is easy to discover that $E_{0}\left(\frac{A}{d},0\right)$ attains the global minimum of the function *V*(*S*, *I*), so *V*(*S*, *I*) > 0. Along system (5), it turns out:

$$\begin{array}{c} \dot{V}{|}_{\left(5\right)}=2A-dS-\frac{A^{2}}{dS}+\frac{\beta AI}{d(1+aI^{2})}-\left(d+\alpha +\mu \left(b,I\right)\right)I. \end{array}$$ (16)

Since *μ*(*b*, *I*) > *μ*_0_ for all *I* ≥ 0, we have

$$\begin{array}{c} \dot{V}{|}_{\left(5\right)}\leq 2A-dS-\frac{A^{2}}{dS}+\frac{\left(\beta A-d\delta_{0}\right)I-da\delta_{0}I^{3}}{d(1+aI^{2})}\leq 0. \end{array}$$ (17)

The equality $\dot{V}\left(S,I\right)=0$ if and only if at $E_{0}\left(\frac{A}{d},0\right)$. By Poincare-Bendixson theorem, theorem 0.4 is obvious.

Let $E(\bar{S},\bar{I})$ be any endemic equilibrium, one can verify that its characteristic equation can be written as

$$\begin{array}{c} \lambda^{2}-\text{tr}\left(J_{E}\right)\lambda +\det\left(J_{E}\right)=0, \end{array}$$ (18)

where

$$\begin{array}{c} \begin{array}{c} \text{tr}\left(J_{E}\right)=-d+\frac{\mu_{1}-\mu_{0}}{{\left(b+\bar{I}\right)}^{2}}b\bar{I}-\left(d+\alpha +\mu \left(b,\bar{I}\right)\right)\frac{2a{\bar{I}}^{2}}{1+a{\bar{I}}^{2}}-\frac{\beta \bar{I}}{1+a{\bar{I}}^{2}},\\ \det\left(J_{E}\right)=\frac{\bar{I}}{{\left(b+\bar{I}\right)}^{2}\left(1+a{\bar{I}}^{2}\right)}\left(b+\bar{I}\right)f^{\prime }\left(\bar{I}\right). \end{array} \end{array}$$ (19)

Obviously, the signs of the eigenvalues are determined by $f^{\prime }\left(\bar{I}\right)$ and tr(*J*_*E*_). From Fig 2, we know that $f^{\prime }\left({\bar{I}}_{1}\right)<0,f^{\prime }\left({\bar{I}}_{2}\right)>0$, so *E*_1_ is a hyperbolic saddle and *E*_2_ is an anti-saddle. *E*_2_ is an attracting node or focus, if tr(*J*_*E*_) < 0; *E*_2_ is a weak focus or a center, if tr(*J*_*E*_) = 0; *E*_2_ is a repelling node or focus, if tr(*J*_*E*_) > 0. So we obtain the following theorem.

**Theorem 0.5**. *For* system (5), *there are two endemic equilibria E*_1_, *E*_2_ *when* $R_{0}<1$ *and* Δ_0_ < 0. *Then the low endemic equilibrium E*_1_ *is a hyperbolic saddle*, *and the higher endemic equilibrium E*_2_ *is an anti-saddle*. *When* $R_{0}>1$ *there is a unique endemic equilibrium*, *which is an anti-saddle*.

## Bifurcation analysis

### Backward bifurcation

**Theorem 0.6**. *When* $R_{0}=1$, system (5) *undergoes backward bifurcation if* $b<\frac{d(\mu_{1}-\mu_{0})}{\beta \delta_{1}}$; *and* system (5) *undergoes forward bifurcation if* $b>\frac{d(\mu_{1}-\mu_{0})}{\beta \delta_{1}}$.

*Proof*. For convenience of the proof, we suppose that the total number of the population is *N*(*t*). System (4) becomes the following system

$$\begin{array}{c} \left\{\begin{array}{c} \frac{dI}{dt}=\frac{\beta (N-I-R)I}{1+aI^{2}}-dI-\alpha I-\left(\mu_{0}+\left(\mu_{1}-\mu_{0}\right)\frac{b}{b+I}\right)I,\\ \frac{dR}{dt}=\left(\mu_{0}+\left(\mu_{1}-\mu_{0}\right)\frac{b}{b+I}\right)I-dR,\\ \frac{dN}{dt}=A-dN-\alpha I. \end{array}\right. \end{array}$$ (20)

Let *V* = (*I*, *R*, *N*)^*T*^, then the disease-free equilibrium is $V_{0}={\left(0,0,\frac{A}{d}\right)}^{T}$ and we can write Eq (20) in vector forms as:

$$\begin{array}{c} \dot{V}=H\left(V\right)\left(V-V_{0}\right), \end{array}$$ (21)

where

$$\begin{array}{c} H\left(V\right)=\left(\begin{array}{ccc} \frac{\beta (N-I-R)}{1+aI^{2}}-d-\alpha -\mu \left(b,I\right) & 0 & 0\\ \mu (b,I) & -d & 0\\ -\alpha & 0 & -d \end{array}\right). \end{array}$$ (22)

Then,

$$\begin{array}{c} H\left(V_{0}\right)=\left(\begin{array}{ccc} \;\delta_{1}\left(R_{0}-1\right) & 0 & 0\\ \mu 1 & -d & 0\\ -\alpha & 0 & -d \end{array}\right). \end{array}$$ (23)

We know that the dominant eigenvalue of *H*(*V*_0_) is zero, if $R_{0}=1$. It is well known that we can decompose a neighborhood of the disease-free state into stable manifold *W*^*s*^ and a center manifold *W*^*c*^. Thus, the dynamic behavior of system (20) can be determined by the flow on the center manifold. We know that zero is a simple eigenvalue and the *W*^*c*^ is tangential to the eigenvector *V*^0^ at *V*_0_. Thus, we can assume that *W*^*c*^ has the following form:

$$\begin{array}{c} W^{c}=\left\{V_{0}+\alpha V^{0}+Z\left(\alpha \right):V^{*}\cdot Z\left(\alpha \right)=0,-\alpha_{0}\leq \alpha \leq \alpha_{0}\right\}, \end{array}$$ (24)

where V* is the dominant left eigenvector of *H*(*V*_0_), *α*_0_ > 0 is a constant, and *Z*: [−*α*_0_, *α*_0_] → Ran(H(V_0_)) satisfies:

$$\begin{array}{c} Z\left(0\right)=\frac{d}{d\alpha }Z\left(0\right)=0. \end{array}$$ (25)

In other words, *W*^*c*^ can be decomposed into two components. The *α* gives the component of the center manifold that lies along the dominant eigenvector; the component that does not lay along the dominant eigenvector can be given by *Z*(*α*). So, V* ⋅ *Z*(*α*) = 0. In order to determine the dynamic behavior of system (20), we just need to see how *α* depends on time *t*.

Let

$$\begin{array}{c} V\left(t\right)=V_{0}+\alpha \left(t\right)V^{0}+Z\left(\alpha \left(t\right)\right), \end{array}$$ (26)

since *W*^*c*^ is an invariant, from Eq (21) we have

$$\begin{array}{cc} \dot{\alpha }\left(t\right)V^{0}+\frac{d}{dt}Z\left(\alpha \left(t\right)\right) & =\dot{V}\left(t\right)\\ & =H\left(V\left(t\right)\right)\left[V\left(t\right)-V_{0}\right]\\ & =H\left(V_{0}+\alpha \left(t\right)V^{0}+Z\left(\alpha \left(t\right)\right)\right)\left[\alpha \left(t\right)V^{0}+Z\left(\alpha \left(t\right)\right)\right], \end{array}$$

Multiplying both sides of the above equation by V* and using the following equations:

$$\begin{array}{c} V^{*}\cdot \frac{d}{dt}Z\left(\alpha \left(t\right)\right)=0,V^{*}H\left(V_{0}\right)=0,V^{*}\cdot V^{0}=1, \end{array}$$ (27)

and *Z*(*α*) = *O*(*α*^2^) we can get that

$$\begin{array}{cc} \dot{\alpha } & =V^{*}\cdot H\left(V_{0}+\alpha V^{0}+Z\left(\alpha \right)\right)\left[\alpha V^{0}+Z\left(\alpha \right)\right]\\ & =V^{*}\cdot H\left(V_{0}+\alpha V^{0}\right)\left[\alpha V^{0}+Z\left(\alpha \right)\right]+O\left(\alpha^{3}\right)\\ & =V^{*}\cdot \left(H\left(V_{0}+\alpha V^{0}\right)-H\left(V_{0}\right)\right)\left[\alpha V^{0}+Z\left(\alpha \right)\right]+O\left(\alpha^{3}\right). \end{array}$$

Note that [*H*(*V*_0_ + *αV*^0^) − *H*(*V*_0_)] is of order *α*, then [*H*(*V*_0_ + *αV*^0^) − *H*(*V*_0_)]*Z*(*α*) is *O*(*α*^3^) and we get that

$$\begin{array}{c} \dot{\alpha }=\alpha V^{*}\cdot \left[H\left(V_{0}+\alpha V^{0}\right)-H\left(V_{0}\right)\right]V^{0}+O\left(\alpha^{3}\right). \end{array}$$ (28)

The sign of this expression for small *α* is what determines whether the disease can invade at the bifurcation point. In the limit, as *α* goes to zero, Eq (28) becomes:

$$\begin{array}{c} \dot{\alpha }=V^{*}\cdot H^{{}^{\prime }}V^{0}\alpha^{2}+O\left(\alpha^{3}\right), \end{array}$$ (29)

where

$$\begin{array}{c} H^{{}^{\prime }}=\frac{dH(V_{0}+\alpha V^{0})}{d\alpha }{|}_{\alpha =0}=\sum_{i}V_{i}^{0}\frac{\partial H}{\partial V_{i}}{|}_{V=V_{0}}, \end{array}$$ (30)

which gives the rate of change of the vector field as the disease invades. Hence, the number

$$\begin{array}{c} h=V^{*}H^{{}^{\prime }}V^{0} \end{array}$$ (31)

determines whether the disease can invade when $R_{0}=1$, and hence gives the sign of the bifurcation. For our system, by computation we can get the V* and *V*^0^ as follows:

$$V^{0}={\left(I^{0},\frac{\mu_{1}}{d}I^{0},-\frac{\alpha }{d}I^{0}\right)}^{T},\;V^{*}=\left(\frac{1}{I^{0}},0,0\right),$$

and

$$H^{{}^{\prime }}=H_{1}^{{}^{\prime }}I^{0}+H_{2}^{{}^{\prime }}\frac{\mu 1}{d}I^{0}-H_{3}^{{}^{\prime }}\frac{\alpha }{d}I^{0},$$

where

$$\begin{array}{c} H_{1}^{{}^{\prime }}=\left(\begin{array}{ccc} -\beta +\frac{\mu_{1}-\mu_{0}}{b} & 0 & 0\\ -\frac{\mu_{1}-\mu_{0}}{b} & 0 & 0\\ \;\;\;\;\;\;\;\;\;0 & 0 & 0 \end{array}\right),H_{2}^{{}^{\prime }}=\left(\begin{array}{ccc} -\beta & 0 & 0\\ \;\;0 & 0 & 0\\ \;\;0 & 0 & 0 \end{array}\right),H_{3}^{{}^{\prime }}=\left(\begin{array}{ccc} \;\;\beta & 0 & 0\\ \;\;0 & 0 & 0\\ \;\;0 & 0 & 0 \end{array}\right). \end{array}$$ (32)

Then we can get that

$$\begin{array}{ll} h & =V^{*}H^{{}^{\prime }}V^{0}\\ & =\left(\frac{\mu_{1}-\mu_{0}}{b}-\frac{\beta \left(d+\alpha +\mu_{1}\right)}{d}\right)I^{0}. \end{array}$$ (33)

According to [27], we know that system (5) undergoes backward bifurcation, when *h* > 0, i.e., $b<\frac{d(\mu_{1}-\mu_{0})}{\beta \delta 1}$; and system (5) undergoes forward bifurcation, when *h* < 0, i.e., $b>\frac{d(\mu_{1}-\mu_{0})}{\beta \delta 1}$.

**Proposition 0.7**. *When* $R_{0}$ *passes through* $R_{0}^{c}$ *and* tr(I*) ≠ 0, system (5) *with a* → 0 *undergoes a saddle-node bifurcation*. *When* $R_{0}=R_{0}^{c}$, E* *is a saddle-node if* tr(I*) ≠ 0, and E* *is a cusp if* tr(I*) = 0.

*Proof*. As *a* → 0, Δ_0_(*a*) → Δ_0_(0). Δ_0_(0) = 0 means that $R_{0}=R_{0}^{c}$ and the two endemic equilibria *E*_1_ and *E*_2_ coalesce at E*. Two eigenvalues of Jacobian matrix *J*(E*) are 0 and tr(I*) for system (5).

If tr(I*) ≠ 0, we can linearize system (5) at the E* and diagonalize the linear part. Then we can get the following form

$$\begin{array}{c} \left\{\begin{array}{c} \dot{X}=\frac{\beta^{2}\left(\mu_{0}-\mu_{1}\right)\left(b\beta -d\right)}{{\left(b+I^{*}\right)}^{3}\left|T\right|}X^{2}+XO\left(|Y|\right)+{O(|Y|}^{2}{,|X,Y|}^{3})\\ \dot{Y}=\text{tr}\left(I^{*}\right)Y+{O(|X,Y|}^{2}) \end{array}\right. \end{array}$$ (34)

Where *T* is the non-singular transformation to diagonalize the linear part. Since $b<\frac{d}{\beta }$, E* is a saddle-node if tr(I*) ≠ 0. Combined with Theorem 0.1, system (5) undergoes saddle-node bifurcation when $R_{0}$ passes through the critical value $R_{0}^{c}$, as *a* → 0. If tr(I*) = 0, E* is a cusp and we will prove it in the next section.

Based on the above analysis, we know that system (5) undergoes some bifurcation. In order to consider the impact of hospital bed number and the incidence rate on the dynamics of the model, we will chose *b* and *β* as bifurcation parameters to describe these bifurcations. The basic production number $R_{0}=1$ defines a straight line *C*_0_ in the (*β*, *b*) plane,

$$C_{0}:\beta =\frac{d\delta_{1}}{A}.$$

**Fig 3:**
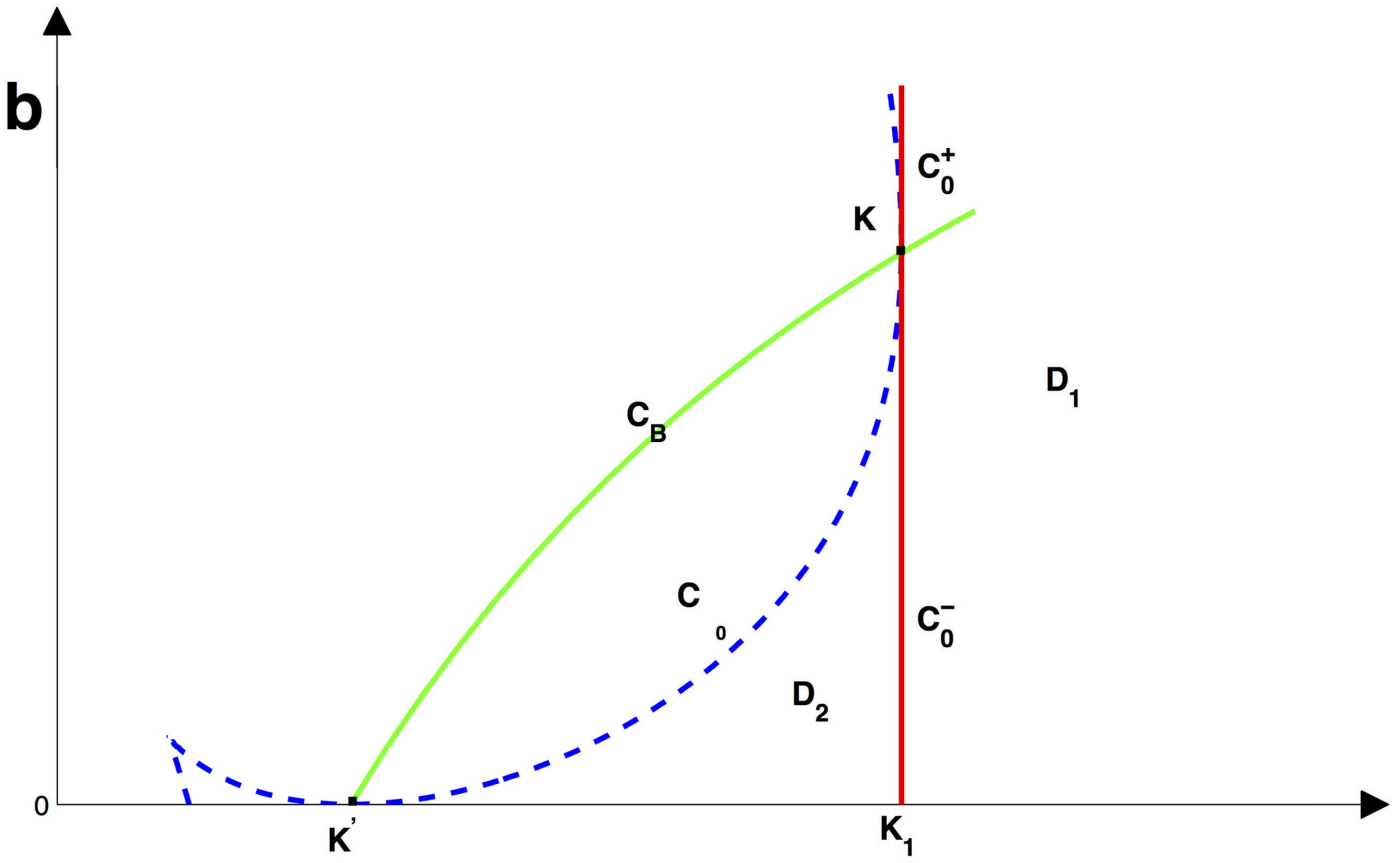
The bifurcation curves in (*β*, *b*) for system (5) when *a* ≠ 0.

C=0 also defines one branch of the hyperbolic *C*_B_ (see Fig 3),

$$C_{B}:b=f_{C}\left(\beta \right)=\frac{\beta A-d\delta_{0}}{\beta \delta_{1}}.$$

The branch of *C*_B_ intersects with *C*_0_ at the point $K(\frac{d\delta_{1}}{A},\frac{A(\mu_{1}-\mu_{0})}{\delta_{1}^{2}})$ and with *β*—axis at the point $K^{{}^{\prime }}\left(\frac{d\delta_{0}}{A},0\right)$. It is easily found that $f_{C}$ is an increasing convex function of *β* in the first quadrant. Let

$$\begin{array}{c} \begin{array}{cc} & C_{0}^{+}=\left\{\left(\beta ,b\right)|\beta =\frac{d\delta_{1}}{A},b>\frac{A(\mu_{1}-\mu_{0})}{\delta_{1}^{2}}\right\},\\ & C_{0}^{-}=\left\{\left(\beta ,b\right)|\beta =\frac{d\delta_{1}}{A},b<\frac{A(\mu_{1}-\mu_{0})}{\delta_{1}^{2}}\right\}, \end{array} \end{array}$$

then $C_{0}=C_{0}^{+}\cup C_{0}^{-}\cup K$. Here $C_{0}^{+}$ and $C_{0}^{-}$ are two branches of *C*_0_ joint at point *K*.

Define the curve Δ_0_(*β*, *b*) = 0 as $C_{\Delta_{0}}$, one can verify that

$$\begin{array}{ccc} & \Delta_{0}\left(K^{{}^{\prime }}\right)=0, & \Delta_{0}\left(K\right)=0,\\ & \frac{\partial b}{\partial \beta }{|}_{K^{{}^{\prime }}}=0, & \frac{\partial b}{\partial \beta }{|}_{K}=\pm \infty . \end{array}$$

Hence, the curve $C_{\Delta_{0}}$ is tangent to the curve *C*_0_ at the point *K* and the *β*—axis at the point *K*′ when $\frac{d\delta_{0}}{A}<\beta <\frac{d\delta_{1}}{A}$.

If $\beta =\frac{d\delta_{1}}{A}$,

$$\begin{array}{c} \Delta_{0}\left(\beta ,b\right)=\Delta_{0}\left(b\right)=-\frac{3d^{4}{\left(b\delta_{1}^{2}+A\left(\delta_{0}-\delta_{1}\right)\right)}^{2}g\left(b\right)}{A^{4}}, \end{array}$$

where

$$\begin{array}{c} g\left(b\right)=A^{2}a^{2}\delta_{1}^{2}b^{2}-2Aa\delta_{0}\delta_{1}^{2}b-\delta_{0}\left(4A^{2}a\left(\delta_{0}-\delta_{1}\right)-\delta_{0}\delta_{1}^{2}\right). \end{array}$$

Denote the discrimination of *g*(*b*) = 0 as $\Delta_{2}=16A^{4}a^{3}\delta_{1}^{2}\delta_{0}\left(\delta_{0}-\delta_{1}\right)<0$. Hence the equation Δ_0_(*b*) = 0 has a unique real solution, $b=\frac{A(\mu_{1}-\mu_{0})}{\delta_{1}^{2}}$, which means that *K* is the only point at which the curve $C_{\Delta_{0}}$ is tangent to the curve *C*_0_.

If *b* = 0,

$$\begin{array}{c} \Delta_{0}\left(\beta ,0\right)=\Delta_{0}\left(\beta \right)=-3\delta_{0}{\left(d\delta_{0}-\beta A\right)}^{2}g_{1}\left(\beta \right), \end{array}$$

where

$$\begin{array}{c} g_{1}\left(\beta \right)=\delta_{0}\beta^{2}+4Aad\beta -4a\delta_{0}d^{2}. \end{array}$$

Through computing, we find that equation Δ_0_(*β*, 0) = 0 has three real solutions

$$\begin{array}{c} \beta_{0}=\frac{d\delta_{0}}{A},\;\;\;\beta_{1,2}=\frac{-2adA\pm 2d\sqrt{A^{2}a^{2}+a\delta_{0}^{2}}}{\delta_{0}}. \end{array}$$

It is easy to verify *β*_0_ > *β*_1_, so the curve $C_{\Delta_{0}}$ will not intersect with the abscissa axis when $\beta \in (\frac{d\delta_{0}}{A},\frac{d\delta_{1}}{A}]$.

For the curve *C*_*B*_, note

$$\begin{array}{c} \begin{array}{ccc} \Delta_{0}\left(\beta ,\frac{\beta A-d\delta_{0}}{\beta \delta_{1}}\right) & = & \frac{12{\left(ad\left(\beta A-d\delta_{0}\right)/\beta +\beta \delta_{0}\right)}^{3}\left(\beta A-d\delta_{0}\right)\left(d\delta_{1}-\beta A\right)}{\beta \delta_{1}}\\ & & +\frac{81a^{2}d^{2}\delta_{0}^{2}{\left(\beta A-d\delta_{0}\right)}^{2}{\left(d\delta_{1}-\beta A\right)}^{2}}{\beta^{2}\delta_{1}^{2}}. \end{array} \end{array}$$ (35)

Obviously, if $\beta \in (\frac{d\delta_{0}}{A},\frac{d\delta_{1}}{A}]$, then $\Delta_{0}\left(\beta ,\frac{\beta A-d\delta_{0}}{\beta \delta_{1}}\right)>0$. Hence, the curve *C*_*B*_ is located above the curve $C_{\Delta_{0}}$ for $\beta \in (\frac{d\delta_{0}}{A},\frac{d\delta_{1}}{A}]$.

Based on the above the discussion and Theorem 0.1, if we define

$$\begin{array}{c} \begin{array}{c} D_{1}=\left\{\left(\beta ,b\right)|\beta >\frac{d\delta_{1}}{A},b>0\right\},\\ D_{2}=\left\{\left(\beta ,b\right)|\frac{d\delta_{0}}{A}<\beta <\frac{d\delta_{1}}{A},0<b<\frac{A(\mu_{1}-\mu_{0})}{\delta_{1}^{2}},\Delta_{0}\left(\beta ,b\right)<0\right\}, \end{array} \end{array}$$

then there is one endemic equilibria in the region *D*_1_ and two endemic equilibria in the region *D*_2_. System (5) undergoes saddle-node bifurcation on the cure $C_{\Delta_{0}}$ when $\beta \in (\frac{d\delta_{0}}{A},\frac{d\delta_{1}}{A}]$. The backward bifurcation occurs on the $C_{0}^{-}$ and forward bifurcation occurs on the $C_{0}^{+}$. The pitchfork bifurcation occurs when transversally passing through the curve *C*_0_ at the point *K*. Especially, if *a* = 0, system (5) has a semi-hyperbolic node of codimension 2 at the point *K* and we can solve *b* in term of *β* from Δ_0_(*β*, *b*) = 0,

$$\begin{array}{c} b=f_{\Delta }^{\pm }\left(\beta \right)=\frac{\beta A\left(\delta_{1}-\delta_{0}\right)+\delta_{0}\left(d\delta_{1}-\beta A\right)\pm 2\sqrt{\beta A\delta_{0}\left(\delta_{1}-\delta_{0}\right)\left(d\delta_{1}-\beta A\right)}}{\beta \delta_{1}^{2}}. \end{array}$$ (36)

Now, we discuss the Hopf bifurcation of system (5). It follows from Eq (18) that if Hopf bifurcation occurs at one endimic equilibrium $E(\bar{S},\bar{I})$, we have tr(*J*_*E*_) = 0. Note that from Eq (19) we can rewrite tr(*J*_*E*_) as

$$\text{tr}\left(J_{E}\right)=-\frac{h_{1}\left(\bar{I}\right)}{{\left(b+\bar{I}\right)}^{2}{\left(1+a{\bar{I}}^{2}\right)}^{2}},$$

where

$$\begin{array}{c} h_{1}\left(\bar{I}\right)=b_{4}{\bar{I}}^{4}+b_{3}{\bar{I}}^{3}+b_{2}{\bar{I}}^{2}+b_{1}\bar{I}+b^{2}d, \end{array}$$ (37)

with

$$\begin{array}{c} \begin{array}{c} b_{4}=a\left(3d+2\mu_{0}+2\alpha \right),\\ b_{3}=a\left(6bd+3b\mu_{0}+b\mu_{1}+4b\alpha \right)+\beta ,\\ b_{2}=a\left(3b^{2}d+2b^{2}\mu_{1}+2b^{2}\alpha \right)+2b\beta +d,\\ b_{1}=b\left(b\beta +2d+\mu_{0}-\mu_{1}\right). \end{array} \end{array}$$

Here, $h_{1}\left(\bar{I}\right)$ is a quartic equation. Since *b*_2_, *b*_3_ and *b*_4_ are non-negative, if $b>\frac{\mu_{1}-\mu_{0}-2d}{\beta }$, *h*_1_(*I*) > 0, in order to make sure that *h*_1_(*I*) = 0 has a positive root, we must have $b<\frac{\mu_{1}-\mu_{0}-2d}{\beta }$. This means sufficient number of hospital beds excludes the possibility of the disease oscillation. From the expression of $h_{1}\left(\bar{I}\right)$ in Eq (37), it is not an easy task to study the Hopf bifurcation from the polynomial Eq (37), we will study a simple case when *a* = 0, and give the simulations to explore the case when *a* is small.

When *a* = 0, the polynomial Eq (37) is reduced to

$$h_{1}\left(\bar{I}\right)=\beta {\bar{I}}^{3}+\left(d+2b\beta \right){\bar{I}}^{2}+b_{1}\bar{I}+b^{2}d.$$

One can verify the following lemma

**Lemma 0.8**. *For any positive equilibrium*, *if* $b\geq \frac{\mu_{1}-\mu_{0}-2d}{\beta }$, *we alway have* tr(*J*_*E*_)<0.

**Fig 4:**
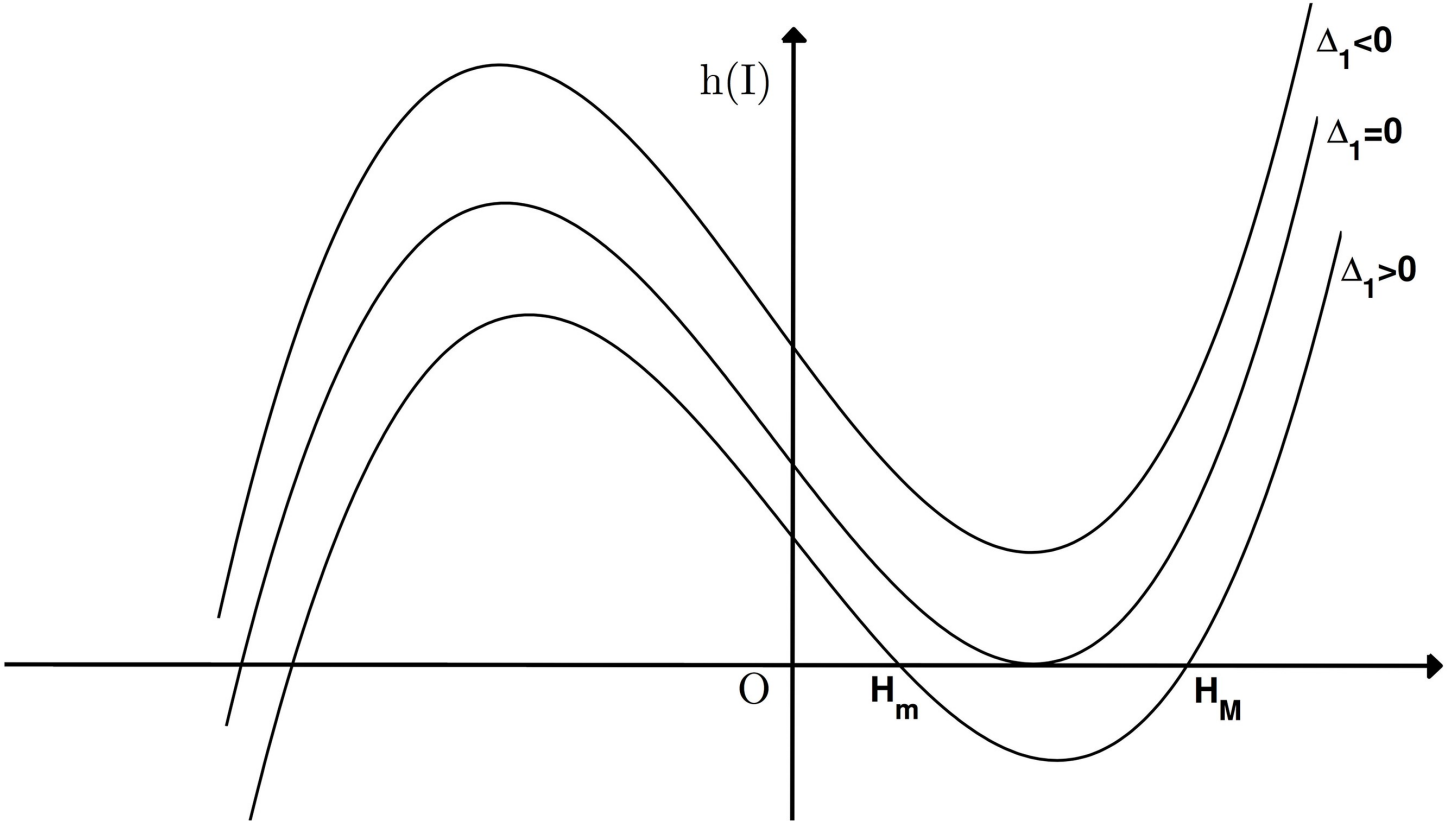
Graph of *h*(*I*) with different signs of Δ_1_ when *b*_1_ < 0. When *I*_2_ = *H*_*m*_, *I*_2_ = *H*_*M*_ or *I*_2_ = *H*_*M*_ = *H*_*m*_, Hopf bifurcation occurs. BT bifurcation of codimension 2 occurs when I* = *H*_*m*_ or I* = *H*_*M*_ and BT bifurcation of codimension 3 occurs when I* = *H*_*m*_ = *H*_*M*_.

In order to study Hopf bifurcation and Bogdanov-Takens bifurcation, we will assume that $b<\frac{\mu_{1}-\mu_{0}-2d}{\beta }$. Denote the discrimination of $h_{1}\left(\bar{I}\right)$ as Δ_1_. Since *b*_1_ < 0, function $h_{1}\left(\bar{I}\right)$ must have one negative real root. As shown in Fig 4, It is not difficult to verify function $h_{1}\left(\bar{I}\right)$ has two humps which locate on the different sides of vertical axis, and the maximum is obtained on the left hump, while the minimum is obtained on the right hump.

The number of roots of function $h_{1}\left(\bar{I}\right)$ is determined by the sign of the Δ_1_. When exist, we denote the roots as *H*_*m*_ and *H*_*M*_ with *H*_*M*_ ≥ *H*_*m*_.

**Lemma 0.9**. $\frac{\partial I_{2}}{\partial \beta }>0,\;\;\;\;\forall \beta >0\;\;\;\;and\;\;\;\;\frac{\partial I_{2}}{\partial b}<0,\;\;\;\;\forall b>0$.

*Proof*. From the Eq (9) and the expression of *I*_2_, direct calculation leads to

$$\begin{array}{c} \frac{\partial I_{2}}{\partial \beta }=-\frac{\partial f}{\partial \beta }/\frac{\partial f}{\partial I_{2}}=\frac{d\delta_{0}I+bd\delta_{1}}{\beta \sqrt{\Delta }}>0,\\ \frac{\partial I_{2}}{\partial b}=\frac{1}{2B}\left[-\frac{\partial C}{\partial b}+\frac{1}{\sqrt{\Delta }}\left(C\frac{\partial C}{\partial b}-2B\frac{\partial D}{\partial b}\right)\right]. \end{array}$$

One can find that $\frac{\partial C}{\partial b}=\beta \delta_{1}>0$. We will prove $\frac{\partial I_{2}}{\partial b}<0$ in two cases. If $R_{0}<1$, then $\frac{\partial D}{\partial b}=d\delta_{1}-\beta A>0$. Recall the analysis of the existence of the equilibria we know that the C < 0, so the $\frac{\partial I_{2}}{\partial b}<0$. If $R_{0}>1$, then $\lim_{b\to +\infty }\frac{\partial I_{2}}{\partial b}=0$ and

$$\begin{array}{cc} \frac{\partial I_{2}^{2}}{\partial^{2}b} & =-\frac{\Delta_{0}^{-3/2}}{8B}\left[{\left(\frac{\partial \Delta_{0}}{\partial b}\right)}^{2}-2\Delta_{0}\frac{\partial^{2}\Delta_{0}}{\partial^{2}b}\right]\\ & =2\beta^{2}A\left(\mu_{1}-\mu_{0}\right)\left(\beta A-d\delta_{1}\right)>0, \end{array}$$

so the $\frac{\partial I_{2}\left(b\right)}{\partial b}$ is an increasing function of *b* with supremum 0 in the (0, +∞), so for ∀*b* > 0 $\frac{\partial I_{2}\left(b\right)}{\partial b}<0$.

**Theorem 0.10**. *For* system (5) *with a* = 0, *generic Hopf bifurcation could occur if I*_2_ = *H*_*m*_, *I*_2_ = *H*_*M*_ *or* *I*_2_ = *H*_*m*_ = *H*_*M*_.

*Proof*. We only need to verify the transversality condition. Let *γ* = tr(*I*_2_)/2 be the real part of the two solutions of Eq (18), when *a* = 0.

If *I*_2_ = *H*_*M*_ or *I*_2_ = *H*_*M*_ = *H*_*m*_, we consider *β* as the bifurcation parameter and fix all other parameters. Then

$$\begin{array}{cc} \frac{d\gamma }{d\beta }{|}_{\beta =\hat{\beta }}= & \frac{1}{2}\left[\frac{\partial \text{tr}(I_{2}\left(\beta \right),\beta )}{\partial I_{2}}\frac{\partial I_{2}\left(\beta \right)}{\partial \beta }+\frac{\partial \text{tr}(I_{2}\left(\beta \right),\beta )}{\partial \beta }\right]{|}_{\beta =\hat{\beta }},\\ \frac{\partial \text{tr}(I_{2}\left(\beta \right),\beta )}{\partial I_{2}}{|}_{\beta =\hat{\beta }}= & -\frac{2h^{{}^{\prime }}\left(H_{M}\right)}{{\left(b+H_{M}\right)}^{2}}<0,\\ \frac{\partial \text{tr}(I_{2}\left(\beta \right),\beta )}{\partial \beta }{|}_{\beta =\hat{\beta }}= & -\frac{H_{M}^{3}+2bH_{M}^{2}+b^{2}H_{M}}{{\left(b+H_{M}\right)}^{2}}<0. \end{array}$$

From Lemma 0.9, we have $\frac{\partial I_{2}}{\partial \beta }{|}_{\beta =\hat{\beta }}>0$, so $\frac{d\gamma }{d\beta }<0$.

If *I*_2_ = *H*_*m*_ or *I*_2_ = *H*_*M*_ = *H*_*m*_, we consider *b* as the bifurcation parameter and fix all the other parameters. Then

$$\begin{array}{cc} \frac{d\gamma }{db}{|}_{b=\hat{b}}= & \frac{1}{2}\left[\frac{\partial \text{tr}(I_{2}\left(b\right),b)}{\partial I_{2}}\frac{\partial I_{2}\left(b\right)}{\partial b}+\frac{\partial \text{tr}(I_{2}\left(b\right),b)}{\partial b}\right]{|}_{b=\hat{b}},\\ \frac{\partial \text{tr}(I_{2}\left(b\right),b)}{\partial b}{|}_{b=\hat{b}}= & -\frac{2h^{{}^{\prime }}\left(H_{m}\right)}{{\left(b+H_{m}\right)}^{2}}>0,\\ \frac{\partial \text{tr}(I_{2}\left(b\right),b)}{\partial b}{|}_{b=\hat{b}}= & \frac{\left(b-H_{m}\right)\left(\mu_{0}-\mu_{1}\right)}{{\left(b+H_{m}\right)}^{2}}<0. \end{array}$$

From Lemma 0.9, we have $\frac{\partial I_{2}}{\partial b}{|}_{b=\hat{b}}<0$, so $\frac{d\gamma }{d\mu_{1}}<0$ (one can verify that *H*_*m*_ < *b*). Then the proof of theorem is completed.

The reason why we choose different parameters to unfold Hopf bifurcation in Theorem 0.10 is that the transversality condition may fail at some point if we focus on one parameter.

In order to verify that Hopf bifurcation occurs in the system, we need to know the type of *E*_2_. If *E*_2_ is a weak focus, Hopf bifurcation can happen, otherwise system does not undergo Hopf bifurcation. Because system (5) is analytic when *a* = 0, *E*_2_ can only be weak focus or center. We can distinguish these two types of singularities by calculating Lyapunov coefficients and verifying it through numerical simulation.

**Fig 5:**
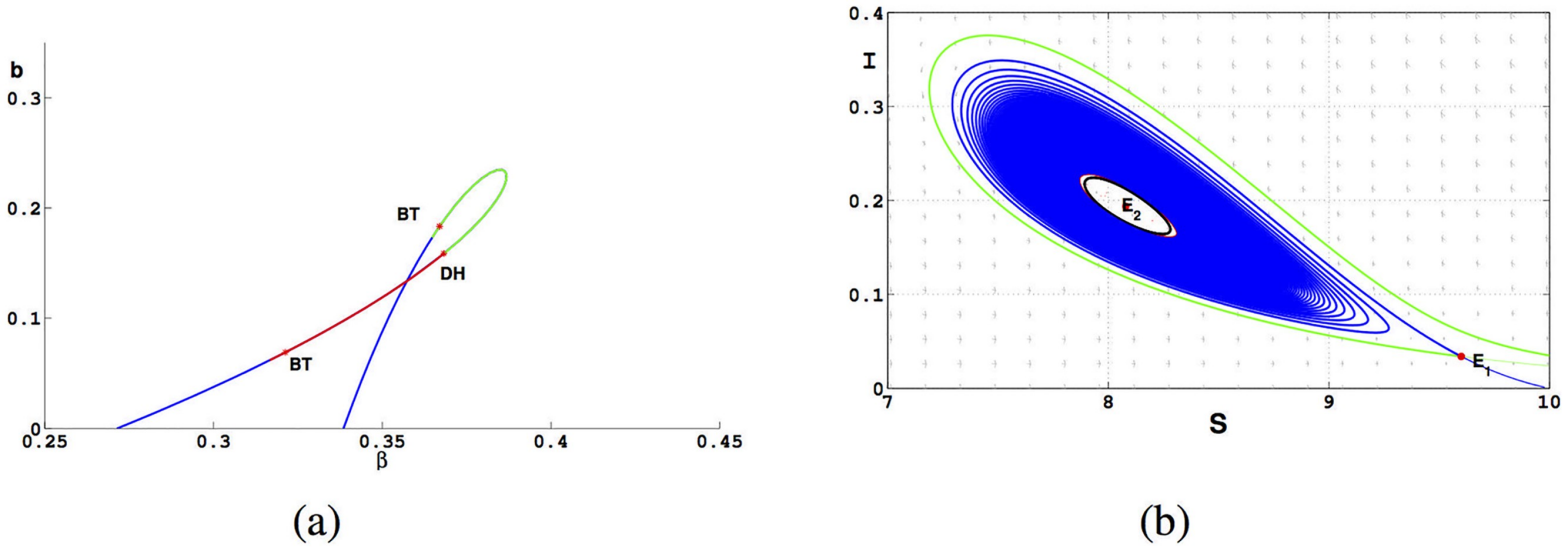
Graphs of Bifurcation curve in parameters plane (*β*, *b*) and the phase trajectory for system (5). (a) Curve *q*(*β*, *b*) = 0. The green curve is supercritical Hopf bifurcation; The red curve is subcritical Hopf bifurcation. *σ*_1_ becomes 0 at the DH point. (b) Two limit cycles bifurcation from the weak focus *E*_2_.

Taking the resultant of *f*(*I*) and $h_{1}\left(\bar{I}\right)$ with respect to *I*, we can get the curve *q*(*β*, *b*) in the space (*β*, *b*), and plot the algebraic curve *q*(*β*, *b*) = 0 by fixing other parameters *A*, *d*, *μ*_1_, *α* and *μ*_0_. Choose *A* = 3, *d* = 0.3, *α* = 0.5, *μ*_0_ = 1.5, *μ*_1_ = 3 and plot *q*(*β*, *b*) = 0 in the plane (*β*, *b*) as shown in Fig 5(a). The green curve (*δ*_1_ < 0) represents supercritical Hopf bifurcation; the red curve corresponding to *δ*_1_ > 0 represents subcritical Hopf bifurcation.

We choose a point(*β*, *b*) = (0.3683, 0.1587) in Fig 5(a) to plot the phase portrait at the point. In Fig 5(b), as *t* → +∞, the trajectory starting at (9, 0.1) spirals outward to the stable limit cycle (red curve) and *E*_2_(8.0794, 0.19363) is stable. Because system (5) is a plane system, there must exist a unstable limit cycle between the stable endemic equilibria and stable limit cycle (black curve). The blue curve in the Fig 5(b) is the unstable manifold of *E*_1_.

### Bogdanov-Takens bifurcation

From Theorem 0.1 we know that the two equilibria *E*_1_ and *E*_2_ coalesce at the equilibria E*(S*, I*) when $R_{0}=R_{0}^{c}$, if *a* = 0, where

$$\begin{array}{c} S^{*}=\frac{A}{d+\beta I^{*}},\\ I^{*}=-\frac{d\left(d+\alpha +\mu_{0}\right)-\beta A+b\beta \left(d+\alpha +\mu_{1}\right)}{2\beta (d+\alpha +\mu_{0})}. \end{array}$$

We can find that det(I*) = 0 in Eq (18) if $R_{0}=R_{0}^{c}$. From Proposition 0.7, we know that E* is a saddle-node point if tr(I*) ≠ 0. If tr(I*) = 0, Eq (18) has a zero eigenvalue with multiplicity 2, which suggests that system (5) may admit a Bogdanov-Takens bifurcation. Then, we give the following theorem.

**Theorem 0.11**. *For* system (5) *with a* = 0, *suppose that* Δ = 0, *h*(I*) = 0 *and h*′(I*) ≠ 0, then E* *is a Bogdanov-Takens point of codimension* 2, *and* system (5) localized at E* *is topologically equivalent to*

$$\begin{array}{c} \left\{\begin{array}{c} \dot{X}=Y,\\ \dot{Y}=X^{2}+Sign\left(h^{{}^{\prime }}\left(I^{*}\right)\right)XY+{O(|X,Y|}^{3}). \end{array}\right. \end{array}$$ (38)

*Proof*. Changing the variables as *x* = *S* − S*, *y* = *I* − I*, then system (5) becomes

$$\begin{array}{c} \left\{\begin{array}{c} \frac{dx}{dt}=-\left(d+\beta I^{*}\right)x-\beta S^{*}y-\beta xy,\\ \frac{dy}{dt}=\beta I^{*}x+\frac{\left(\mu_{1}-\mu_{0}\right)bI^{*}}{{\left(b+I^{*}\right)}^{2}}y+Cy^{2}+\beta xy+{O(|x,y|}^{3}), \end{array}\right. \end{array}$$ (39)

where

$$\begin{array}{c} C=\frac{(\mu_{1}-\mu_{0})b}{{\left(b+I^{*}\right)}^{2}}-\frac{\left(\mu_{1}-\mu_{0}\right)bI^{*}}{{\left(b+I^{*}\right)}^{3}}. \end{array}$$ (40)

tr(I*) = 0 and det(I*) = 0 imply that

$$\begin{array}{c} \begin{array}{cc} \beta^{2}S^{*}I^{*}-{\left(d+\beta I^{*}\right)}^{2}=0, & d+\beta I^{*}=\frac{\left(\mu_{1}-\mu_{0}\right)bI^{*}}{{\left(b+I^{*}\right)}^{2}}, \end{array} \end{array}$$ (41)

so the generalized eigenvectors corresponding to *λ* = 0 of Jacobian matrix in system (5) at the point E* are

$$\begin{array}{c} \begin{array}{cc} V_{1}={\left(-d-\beta I^{*},\beta I^{*}\right)}^{{}^{\prime }}, & V_{2}={\left(1,0\right)}^{{}^{\prime }}. \end{array} \end{array}$$ (42)

Let *T* = (*T*_*ij*_)_2×2_ = (*V*_1_, *V*_2_), then under the non-singular linear transformation

$$\begin{array}{c} \left(\begin{array}{c} x\\ y \end{array}\right)=T\left(\begin{array}{c} X\\ Y \end{array}\right), \end{array}$$

where |*T*| = −βI* < 0. System (39) becomes

$$\begin{array}{c} \left\{\begin{array}{c} \dot{X}=Y+a_{11}X^{2}+\beta XY,\\ \dot{Y}=a_{21}X^{2}+d\beta XY+{O(|X,Y|}^{3}), \end{array}\right. \end{array}$$ (43)

here

$$\begin{array}{c} \begin{array}{c} a_{11}=-\frac{\left(d+\beta I^{*}\right)\beta^{2}I^{*}-\left(\frac{(\mu_{1}-\mu_{0})b}{{\left(b+I^{*}\right)}^{2}}-\frac{\left(\mu_{1}-\mu_{0}\right)bI^{*}}{{\left(b+I^{*}\right)}^{3}}\right)\beta^{2}I^{{*}^{2}}}{\beta I^{*}},\\ a_{21}=\frac{\beta I^{*}\left(\beta I^{*}+d\right)\left(b\beta -d\right)}{b+I^{*}}. \end{array} \end{array}$$

Using the near-identity transformation

$$\begin{array}{c} u=X-\frac{a_{12}X^{2}}{2},\;\;\;\;\;v=Y+a_{11}X^{2}, \end{array}$$ (44)

and rewrite *u*, *v* into *X*, *Y*, we obtain

$$\begin{array}{c} \left\{\begin{array}{c} \dot{X}=Y+{O(|X,Y|}^{3}),\\ \dot{Y}=M_{21}X^{2}+M_{22}XY+{O(|X,Y|}^{3}). \end{array}\right. \end{array}$$ (45)

To consider the sign of *M*_21_, note that

$$M_{21}=a_{21}=\frac{\beta I^{*}\left(\beta I^{*}+d\right)\left(b\beta -d\right)}{b+I^{*}}.$$

For system (5), the condition of the existence of endemic equilibrium is $b<\frac{d(\mu_{1}-\mu_{0})}{\beta \delta_{1}}$, hence, *M*_21_ < 0. Then we will determine the sign of *M*_22_ by

$$\begin{array}{ll} M_{22} & =a_{22}+2a_{11}\\ & =d\beta -2\frac{\left(d+\beta I^{*}\right)\beta^{2}I^{*}-\left(\frac{\left(\mu_{1}-\mu_{0}\right)b}{{\left(b+I^{*}\right)}^{2}}-\frac{\left(\mu_{1}-\mu_{0}\right)bI^{*}}{{\left(b+I^{*}\right)}^{3}}\right)\beta^{2}I^{{*}^{2}}}{\beta I^{*}}\\ & =-\frac{\beta h^{{}^{\prime }}\left(I^{*}\right)}{{\left(b+I^{*}\right)}^{2}}. \end{array}$$

If *h*′(I*) ≠ 0, we make a change of coordinates and time and preserve the orientation by time

$$\begin{array}{c} X\to \frac{M_{21}}{a_{22}^{2}}X,\;\;\;\;\;\;Y\to \frac{M_{21}^{2}}{a_{22}^{3}}Y,\;\;\;\;\;\;t\to |\frac{a_{22}}{M_{21}}|t \end{array}$$ (46)

then system (5) is topologically equivalent to the normal form Eq (38).

From Theorem 0.11, we know that if *a* = 0, endemic equilibrium E* is a Bogdanov-Takens point of codimension 2 when Δ = 0, *h*(I*) = 0 and *h*′(I*) ≠ 0. If *h*′(I*) = 0, E* may be a cusp of codimension 3.

In [28], a generic unfolding with the parameters *ε* = (*ε*_1_, *ε*_2_, *ε*_3_) of the codimension 3 cusp singularity is *C*^∞^ equivalent to

$$\begin{array}{c} \left\{\begin{array}{c} \dot{X}=Y,\\ \dot{Y}=\varepsilon_{1}+\varepsilon_{2}Y+\varepsilon_{3}XY+X^{2}-X^{3}Y+{O(|X,Y|}^{4}). \end{array}\right. \end{array}$$ (47)

**Fig 6:**
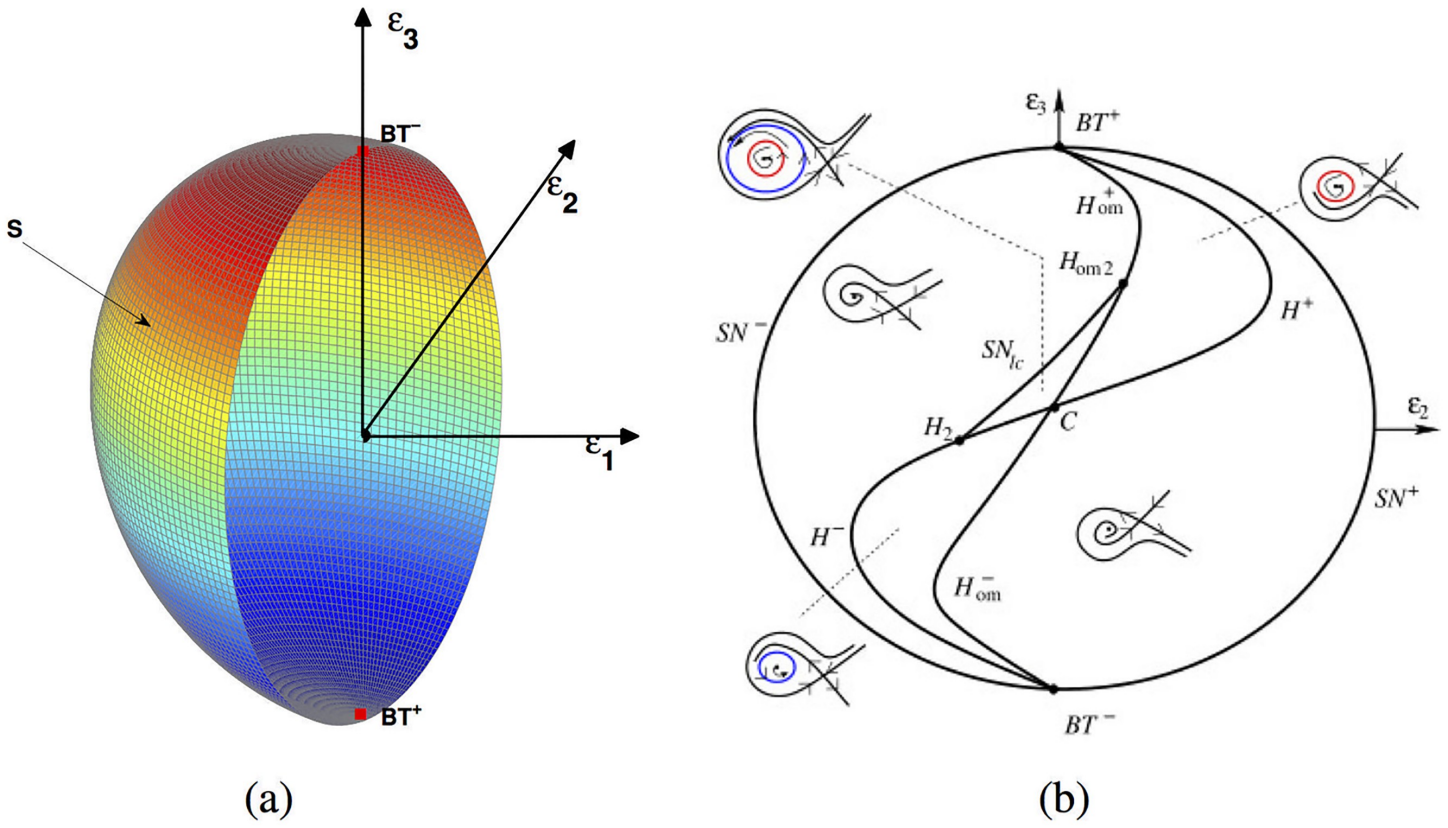
The bifurcation diagram of BT of codimension 3. (a) The parameter space and the trace of the bifurcation diagram on the *S*(*ϵ*_1_ ≤ 0); (b) The sign of the BT is positive if the coefficient of the term *XY* in the norm form is positive, otherwise it is negative [24].

About system (47), we can find that the system has no equilibrium if *ε*_1_ > 0. The plane *ε*_1_ = 0 excluding the origin in the parameter space is saddle-node bifurcation surface. When *ε*_1_ decrease from this surface, the saddle-node point of Eq (47) becomes a saddle and a node. Then the other bifurcation surfaces are situated in the half space *ε*_1_ < 0. They can be visualized by drawing their trace on the half-sphere

$$\begin{array}{c} S=\{\left(\varepsilon_{1},\varepsilon_{2},\varepsilon_{3}\right)|\varepsilon_{1}<0,\varepsilon_{1}^{2}+\varepsilon_{2}^{2}+\varepsilon_{3}^{2}=\sigma^{2}\}, \end{array}$$ (48)

when *σ* > 0 sufficiently small (see Fig 6(a)). We recall that the bifurcation set is a ‘cone’ based on its trace with *S*.

In Fig 6(b), trace on the *S* which consists of 3 curves: a curve *H*_*om*_ of homoclinic bifurcation, a *H* of Hopf bifurcation and *SN*_*lc*_ of double limit cycle bifurcation. The curve *SN*_*lc*_ include two points *H*_2_ and *H*_*om*2_ and the curve *SN*_*lc*_ tangent to the curves *H* and *H*_*om*_. The curves *H* and *H*_*om*_ touch the $\partial S=\{\left(\varepsilon_{1},\varepsilon_{2},\varepsilon_{3}\right)|\varepsilon_{1}=0,\varepsilon_{2}^{2}+\varepsilon_{3}^{2}=\sigma^{2}\}$ with a first-order tangency at the points *BT*^+^ and *BT*^−^. In the neighbourhood of the *BT*^+^ and *BT*^−^, one can find the unfolding of the cusp-singularity of codimension 2. For system (47), there exists an unstable limit cycle between *H* and *H*_*om*_ near the *BT*^+^ and an unique stable limit cycle between *H* and *H*_*om*_ near the *BT*^−^. In the curved triangle *CH*_2_*H*_*om*2_ the system has two limit cycles, the inner one unstable and the outer one stable. These two limit cycles coalesce when the *ε* crosses over the curve *SN*_*lc*_. On the *SN*_*lc*_ there exists a unique semistable limit cycle. The more interpretation can be found in literature [24, 28].

### Bifurcation diagram and simulation

According to analysis and Theorem 0.11, we know that system (5) undergoes Bogdanov-Takens bifurcation of codimension 2. In this section we will choose the parameters *β* and *b* as bifurcation parameters to present the bifurcation diagram by simulations. In the proof of Theorem 0.11, we make a series of changes of variables and time, so there will be different situations with different signs of *h*′(*I*). According to the positive and negative coefficients of *XY* term in the normal form Eq (47), we denote the Bogdanov-Takens bifurcation of codimension 2 as *BT*^+^ and *BT*^−^ respectively.

**Fig 7:**
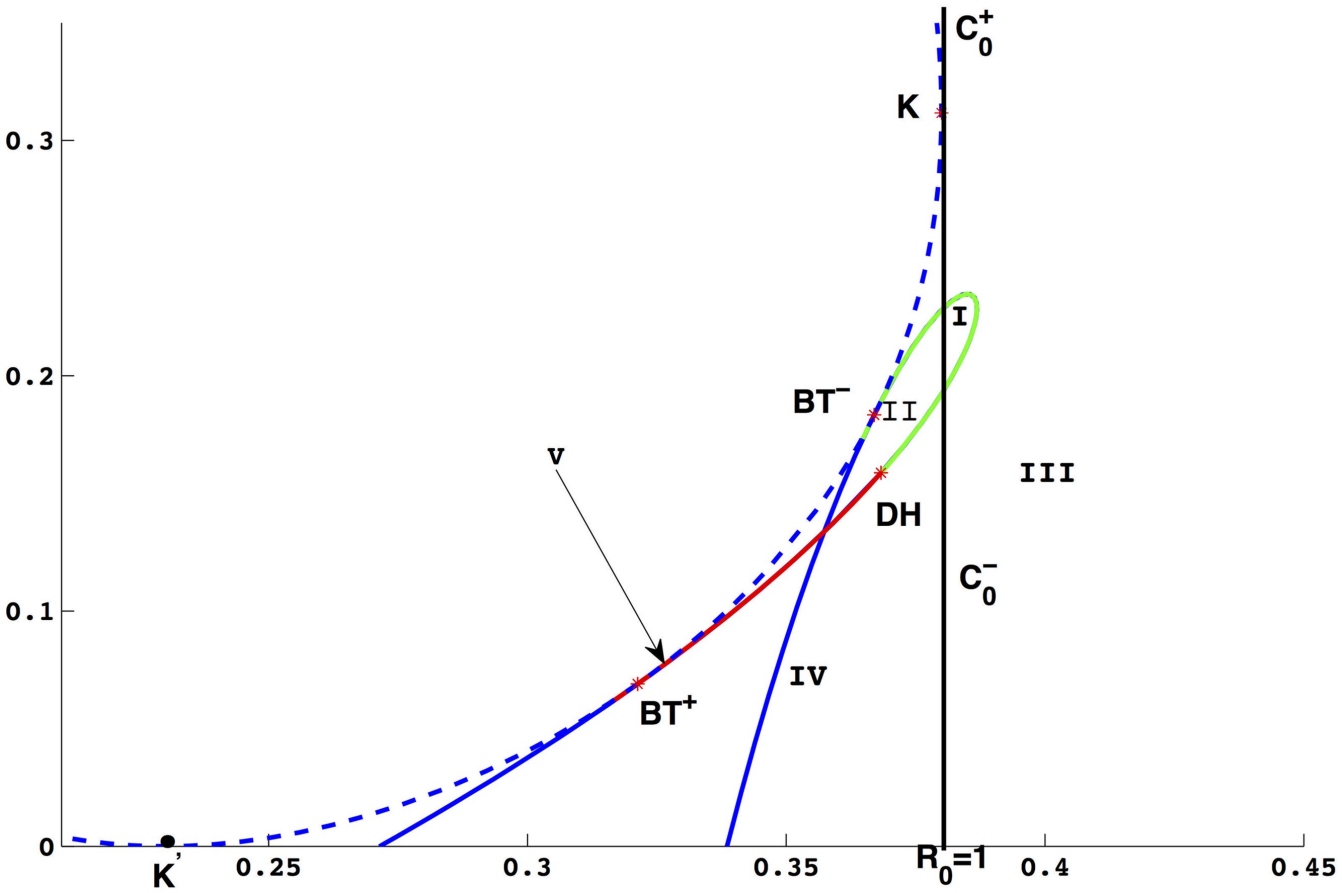
The bifurcation diagram in plane (*β*, *b*). There are two types of Bogdanov-Takens bifurcation, *BT*^+^ and *BT*^−^. The green curve represents supercritical Hopf bifurcation, the red curve represents subcritical Hopf bifurcation. The blue dash (solid) curve represents saddle-node bifurcation (neutral saddle curve).

**Fig 8:**
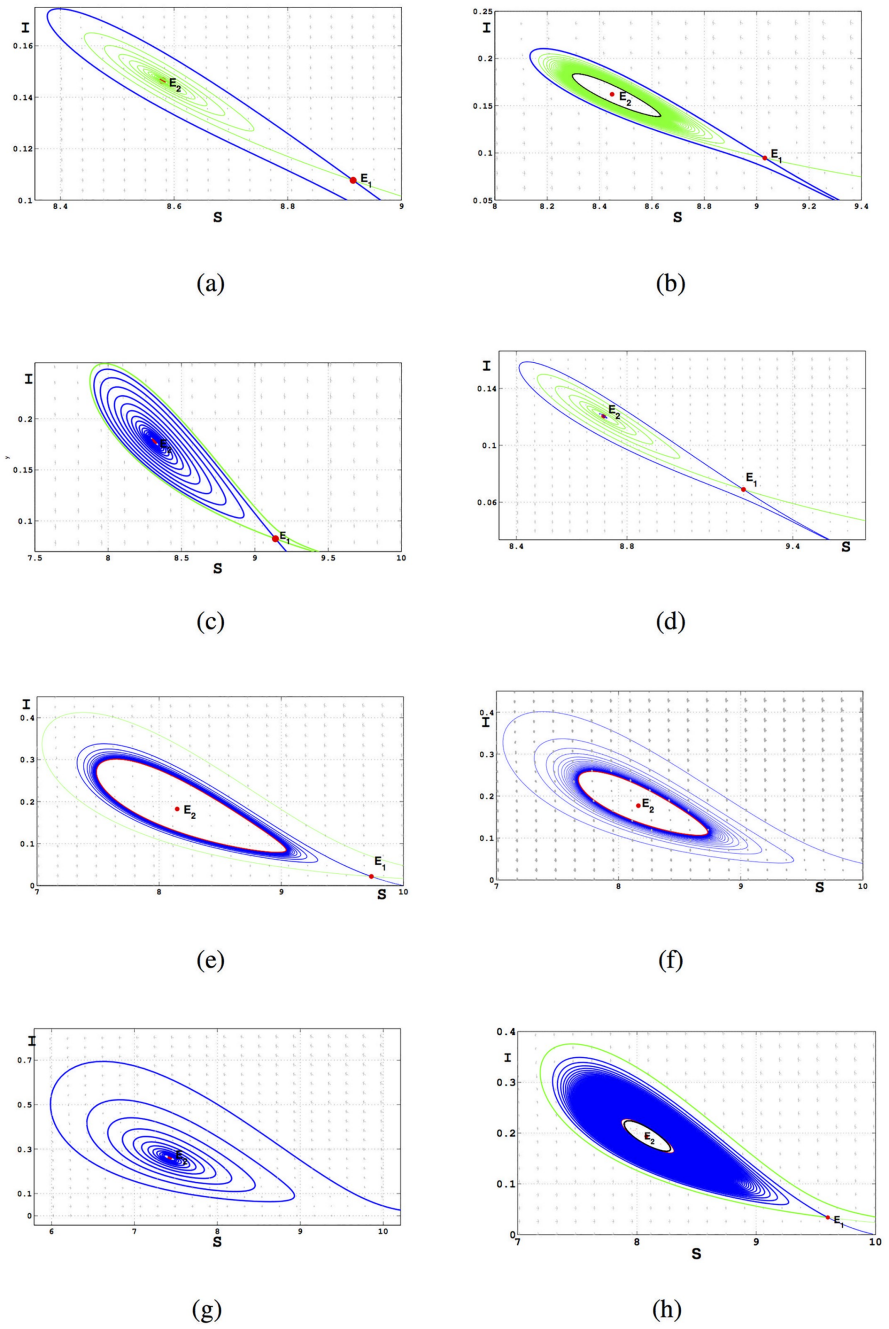
The phase diagram of system (5). The blue curve represents unstable manifold, green curve represents stable manifold. (a) *b* = 0.1, *β* = 0.339; (b) *b* = 0.1, *β* = 0.340; (c) *b* = 0.1, *β* = 0.3415; (d) *b* = 0.18, *β* = 0.367; (e) *b* = 0.18, *β* = 0.3737; (f) *b* = 0.21, *β* = 0.3815; (g) *b* = 0.21, *β* = 0.4; (h) *b* = 0.1587, *β* = 0.3683. In the (b), there is an unstable limit cycle marked black curve near the epidemic equilibrium *E*_2_. In the (e) and (f), there is a stable limit cycle marked red curve. In the (h), we find that there are two limit cycle, the small one is unstable, the another one is stable.

Taking *A* = 3, *d* = 0.3, *α* = 0.5, *μ*_0_ = 1.5, *μ*_1_ = 3, *a* = 0, we find that (*β*, *b*) = (0.367004, 0.183323) satisfying the conditions in Theorem 0.11, then we use (*β*, *b*) to unfold the Bogdanov-Takens bifurcation of codimension 2. By simulation, we obtain the bifurcation diagram in plane (*β*, *b*) shown as Fig 7, the blue dash (solid) curve represents saddle-node bifurcation (neutral saddle), the green (blue solid) curve represents supercritical (subcritical) Hopf bifurcation and the parameter space (*β*, *b*) is divided into differen areas by these bifurcation curves. There are two Bogdanov-Takens bifurcation points, *BT*^−^(0.367004, 0.183323) and *BT*^+^(0.321298, 0.069049). In order to distinguish these two points, we get some phase diagrams of system when *β* and *b* located in different area of (*β*, *b*) shown as Fig 8.

In Fig 8(a), *β*, *b* are located in the area between saddle-node bifurcation and subcritical Hopf bifurcation curve and the epidemic equilibrium *E*_2_ is a unstable focus. In the IV, the phase diagram of system is one of the cases shown as (*b*), (*c*) and (*h*). There is an unstable limit cycle (black curve) near the epidemic equilibrium *E*_2_ in Fig 8(b) and two limit cycles in Fig 8(h) with the inner one unstable and the other one stable. When *β* and *b* are located in II, the phase diagram of system is one of the cases as shown in (*d*) and (*e*) and there is a stable limit cycle in Fig 8(e). When *β* and *b* are located in I or III, the phase portraits are similar to the cases of (*f*) and (*g*), respectively. In the case (*f*), system (5) has a unique epidemic equilibrium and a stable limit cycle.

**Fig 9:**
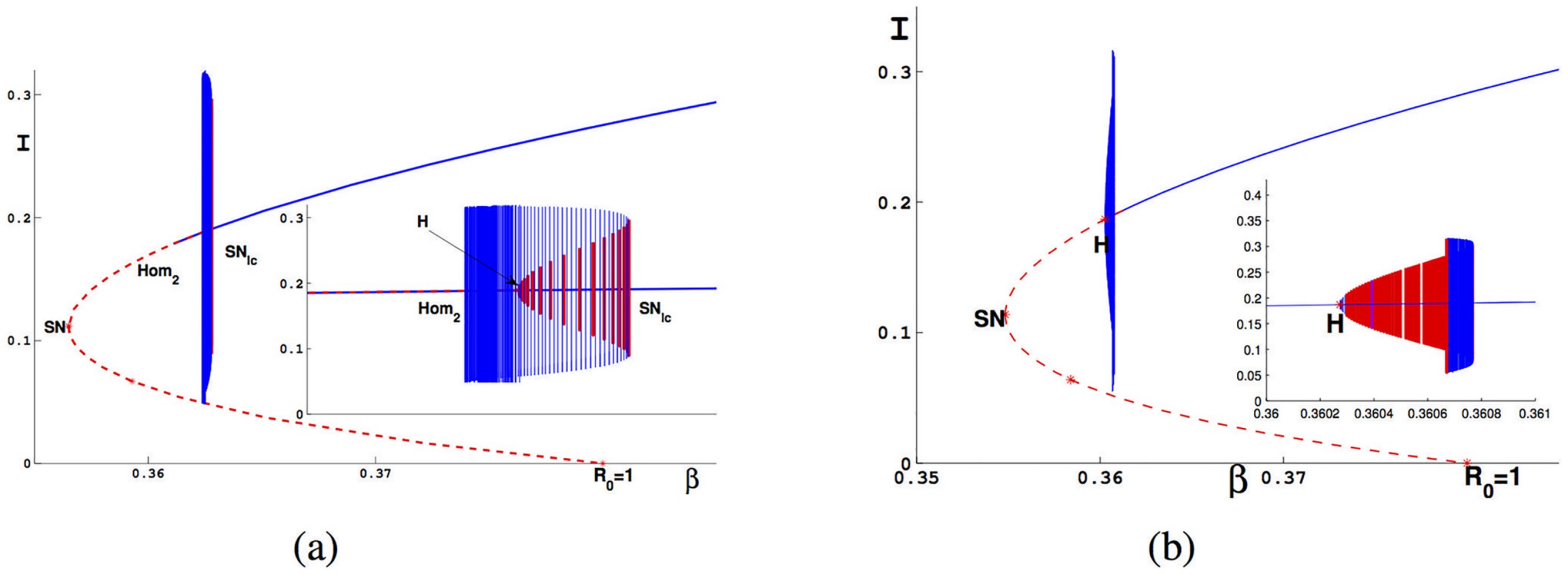
Bifurcation diagram in (*β*, *I*) with different *b*. The red dash(solid) represents unstable epidemic equilibrium(limit cycle). The blue curve represents stable epidemic equilibrium or limit cycle. (a) *b* = 0.145; (b) *b* = 0.14.

In the small neighborhood of *BT*^+^, we know that the unstable limit cycle bifurcating from Hopf bifurcation curve disappears from the homoclinic loop, and from Fig 8(b) and 8(c), we can observe that the homoclinic loops are located in IV. Otherwise, from Fig 8(d) and 8(e), we can obtain that the homoclinic loops are located in II which is in the small neighborhood of *BT*^−^. Hence, the Hopf bifurcation curve and homoclinic loops switch their positions at some point *C*. In order to figure out the relative positions of *C*, *H*_2_ and *Hom*_2_, as shown in Fig 9 we change the value of *β* and plot the bifurcation diagram on (*β*, *b*) with different *b*.

By simulations, we get the case (a) and (b) in Fig 9, we now know that two limit cycles bifurcating from the semi-stable cycle with one disappearing from the Homoclinic loop and the another disappearing from the Hopf bifurcation curve. We therefore obtain $b_{H_{2}}>b_{C}>b_{H_{om2}}$ by the order these two limit cycle vanishing with different values of *b*.

**Fig 10:**
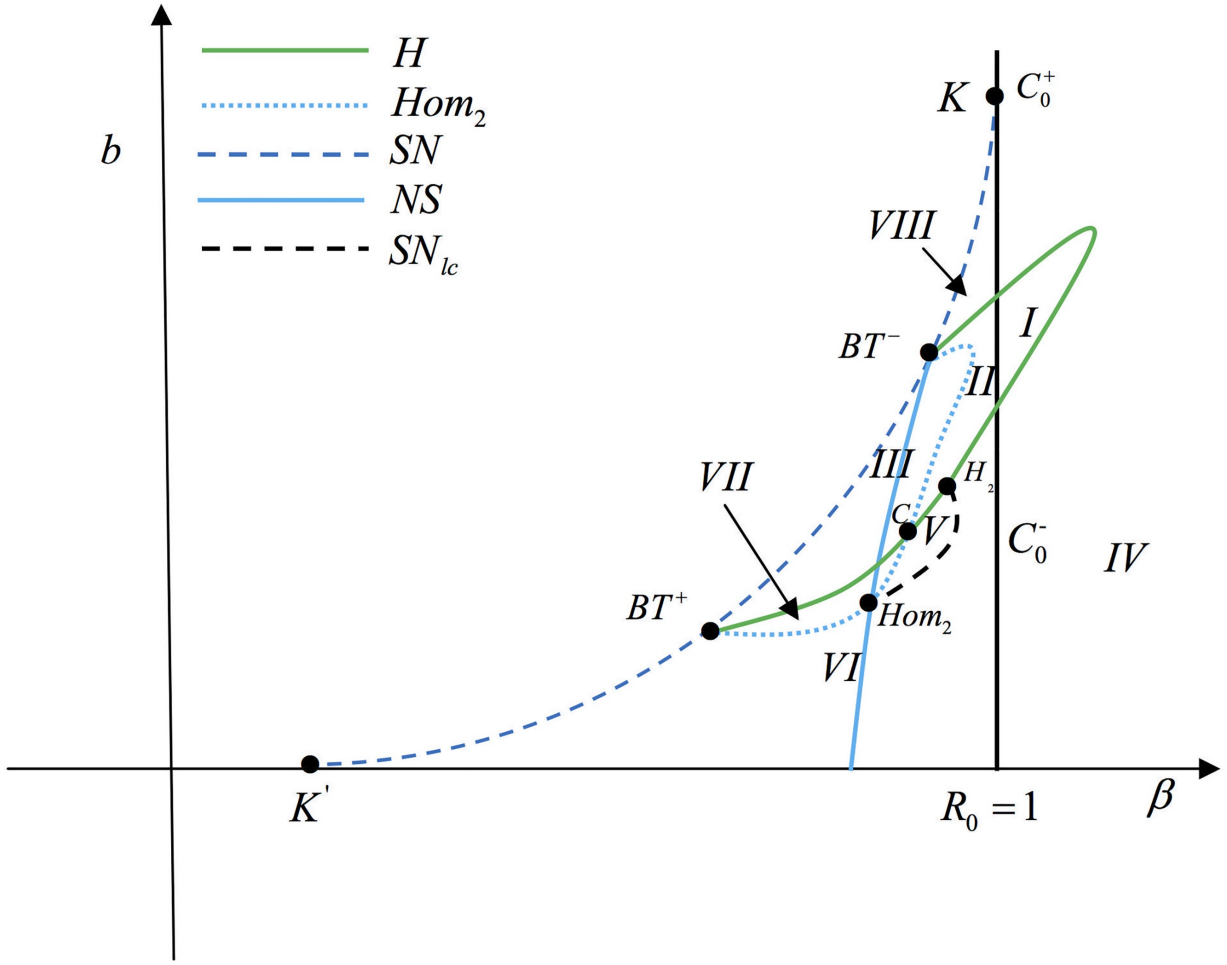
Bifurcation digram near the Bogdanov-Takens.

**Fig 11:**
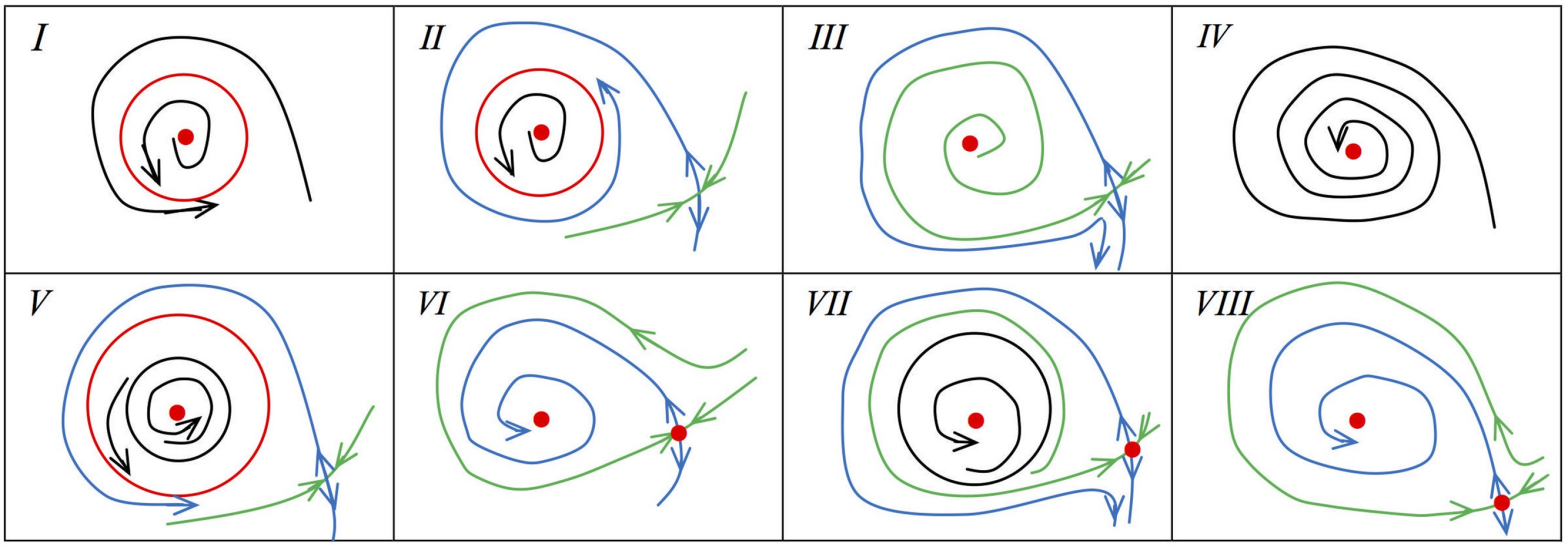
Phase portraits for parameters in different regions of Fig 10.

Then we obtain the bifurcation diagrams of system (5) near the Bogdanov-Takens bifurcation and the phase portraits in some regions of parameters shown as Figs 10 and 11 respectively.

**Fig 12:**
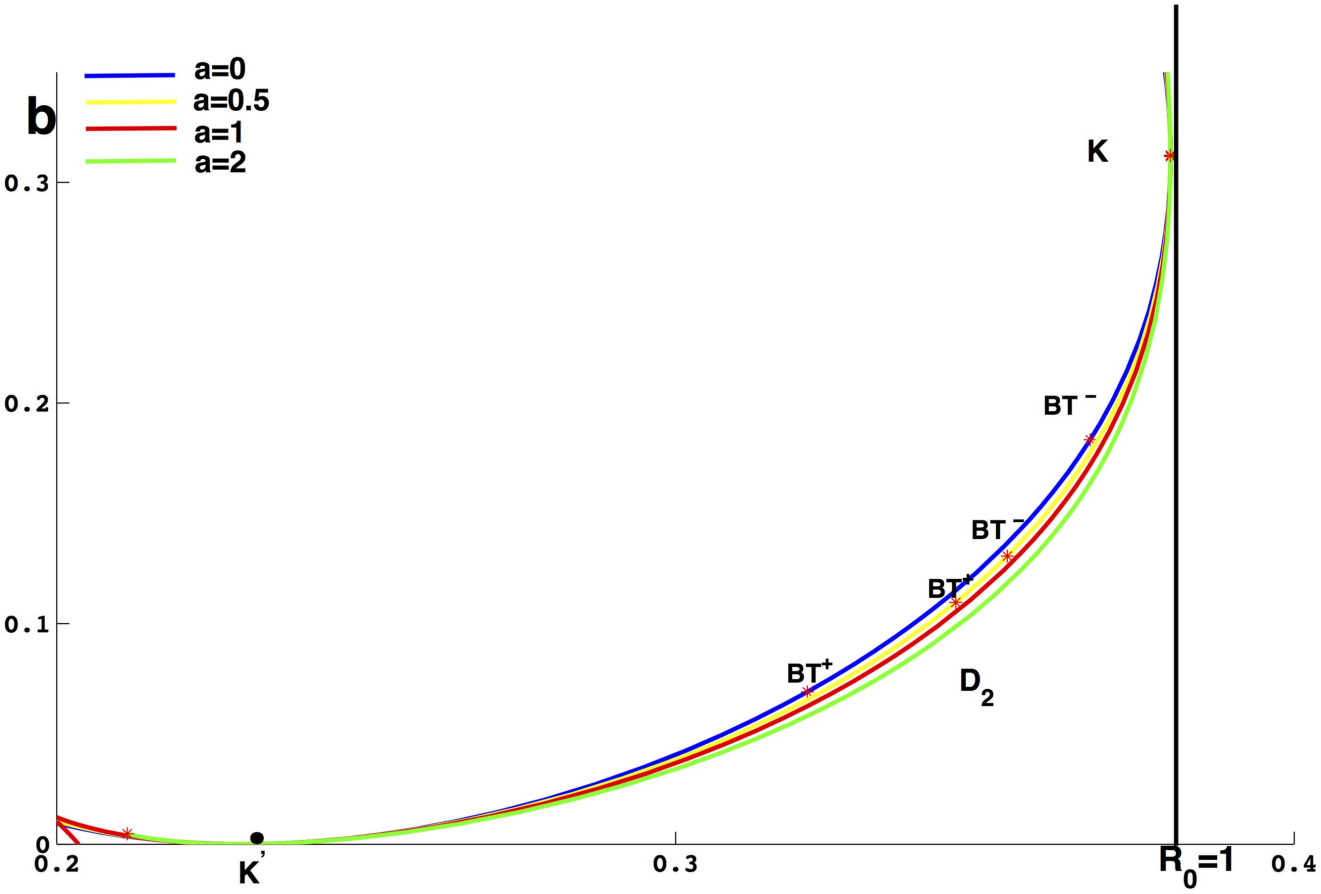
The curve *q*(*β*, *b*) = 0 with different values of *a*. The blue curve, yellow curve, red curve and green curve are drawn according to *a* = 0, *a* = 0.5, *a* = 1 and *a* = 2 respectively.

From the above dynamical analysis, we know that system (5) has complex dynamic behavior even though *a* = 0. For system (5), we also find the same phenomenon by the simulation as shown in the Fig 12 for the case *a* ≠ 0. In the simulation, the parameters excluding *a* are the same as the simulation setting. From Fig 12, we find that the region *D*_2_ and the distance between *BT*^+^ and *BT*^−^ becomes small when *a* becomes lager, which means that choosing *a* as one other bifurcation parameter can unfold the system (5) and system (5) may undergo the Bogdanov-Takens bifurcation of codimension 3.

## Discussion and application

In this paper we consider the SIR model with the nonmonotone incidence rate due to the intervention strategies and nonlinear recovery rate considering the hospitalization conditions.

From Theorem 0.1, we know that system (4) undergoes backward bifurcation. In Theorem 0.3, we get the necessary and sufficient condition of backward bifurcation is $b<\frac{A(\mu_{1}-\mu_{0})}{\delta_{1}^{2}}$ when $R_{0}=1$, which means that we can eliminate the disease if $b>\frac{A(\mu_{1}-\mu_{0})}{\delta_{1}^{2}}$ and $\beta <\frac{d\delta_{1}}{A}$ i.e we need enough number of hospital beds. From the Lemma 0.8, we know that if $b>\frac{\mu_{1}-\mu_{0}-2d}{\beta }$, system (5) will not have periodic solution, and the endemic equilibrium *E*_2_ is stable. We then discuss Hopf bifurcation and BT bifurcation for system (5) and present in details about these bifurcations in the case *a* = 0 and present the bifurcation diagrams in Figs 8 and 10.

**Fig 13:**
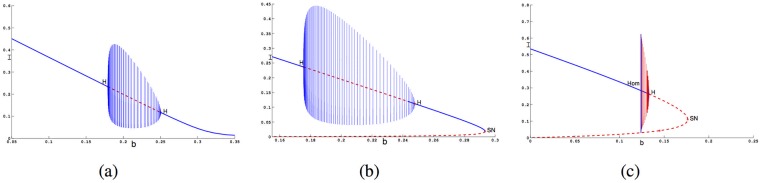
Bifurcation in the plane (*b*, *I*) with different *μ*_1_. *β* = 0.39, *a* = 0. (a) $R_{0}=1.02$; (b) $R_{0}=0.98$; (c) $R_{0}=0.95$.

From the discussion we get Lemma 0.9, which implies that *I*_2_(*b*) is a monotone decreasing function of *b*. Hence, increasing the number of beds can only reduce the number of the total infected individuals, but can not eliminate the diseases as shown in Fig 13(a) if $R_{0}>1$. If $R_{0}<1$, from Fig 3 and Eq (36), we know that if $b>b_{c}=f_{\Delta }^{-}$ we can eliminate the disease, and these rich dynamics finally disappear through the saddle-node bifurcation when *b* = *b*_*c*_ as shown in Figs 7 and 13(b) and 13(c).

**Fig 14:**
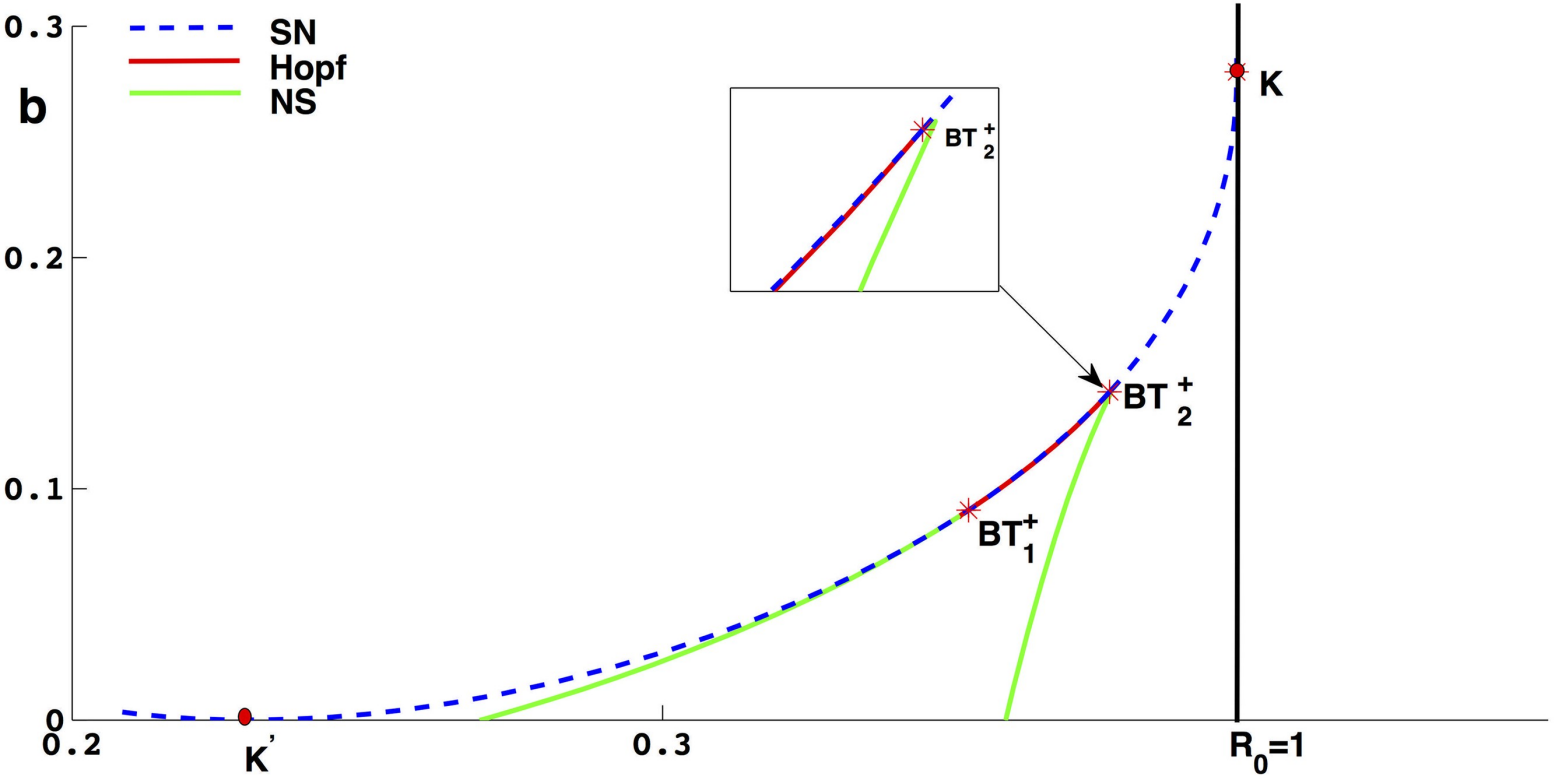
The bifurcation diagram of system (3) in parameters plane (*β*, *b*). *A* = 3, *d* = 0.3, *β* = 0.5, *c* = 0.185, *a* = 0.2, *μ*_1_ = 3.1728627, *μ*_0_ = 1.5, $BT_{1}^{+}\left(0.353073,0.0925301\right),$ $BT_{2}^{+}\left(0.375,0.137406\right)$.

**Fig 15:**
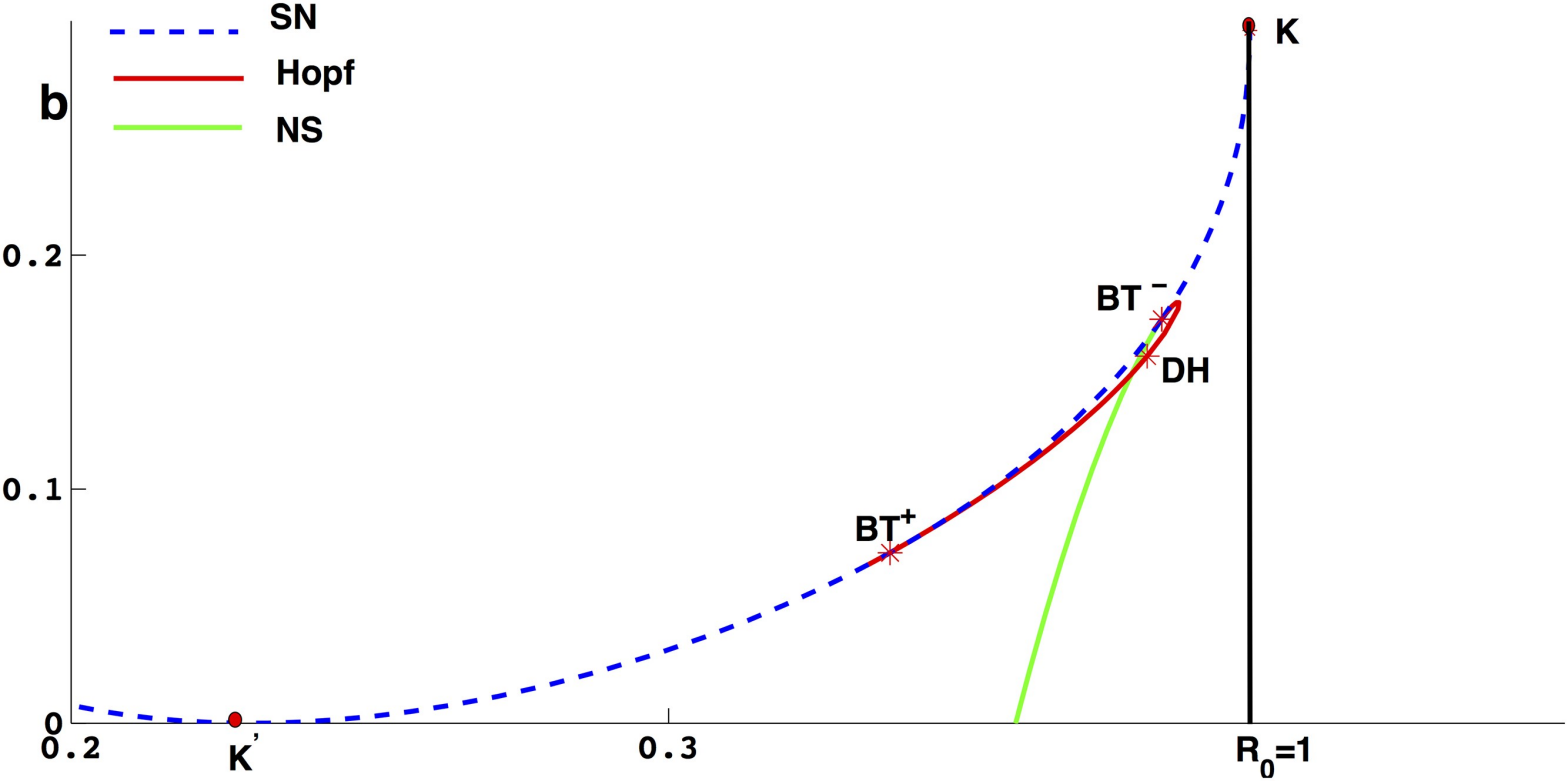
The bifurcation diagram of system (3) in parameters plane (*β*, *b*). *A* = 3, *d* = 0.3, *β* = 0.5, *c* = 0.1, *a* = 0.2, *μ*_1_ = 3.1728627, *μ*_0_ = 1.5, *BT*^+^(0.337066, 0.072821), *BT*^−^(0.382572, 0.172627).

For system (3) with *a* ≠ 0, taking *A* = 3, *d* = 0.3, *β* = 0.5, *a* = 0.2, *μ*_1_ = 3.1728627 and *μ*_0_ = 1.5 we get the bifurcation diagram with different values for *c* as shown as Figs 14 and 15. In Fig 14, the types of BT-bifurcation are the same, however, there are two types of BT bifurcations in the Fig 15.

**Fig 16:**
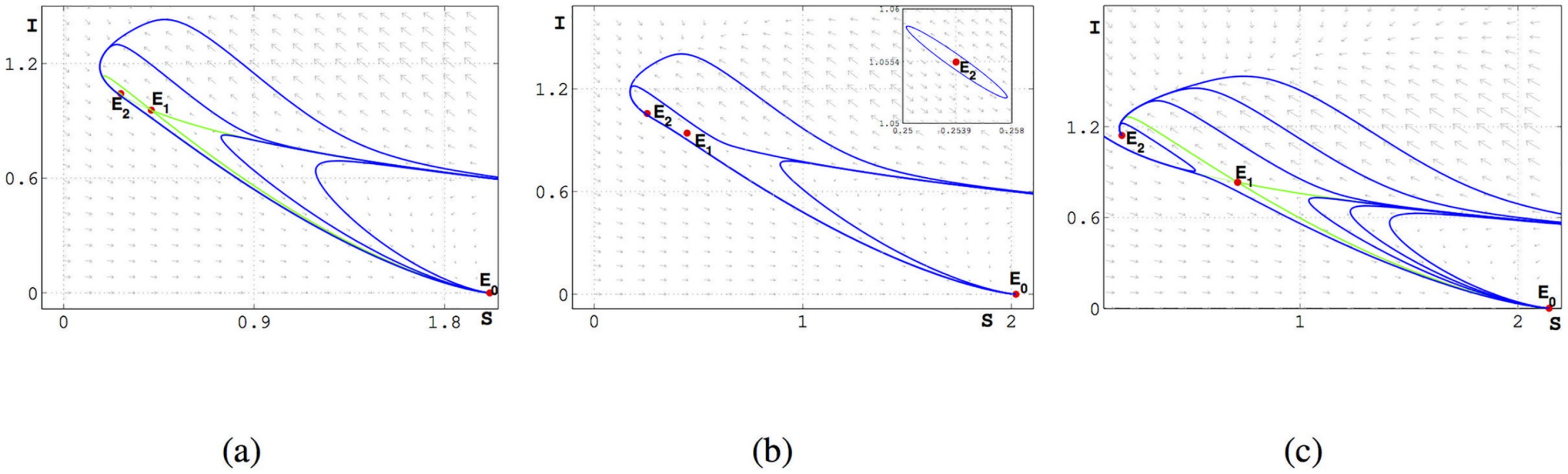
Phase portraits of system (3) in the plane (*S*, *I*) with different *d*. *A* = 3, *β* = 0.375, *α* = 0.5, *μ*_0_ = 0.5, *μ*_1_ = 0.7, *a* = 0.7, *b* = 0.01, *c* = −1.65. (a) *d* = 1.49; (b) *d* = 1.483783; (c) *d* = 1.4. In case (a) (b) and (c), *E*_1_ is always a saddle and *E*_0_ is a stable node. *E*_2_ is a stable node in case (b) and (c), while it is a unstable node in case (a). There is an unstable limit cycle near *E*_2_ in case (b).

In Fig 16, *A* = 3, *β* = 0.375, *α* = 0.5, *μ*_0_ = 0.5 we plot the phase portraits in plane (*S*, *I*) with different *d*, in these cases $\frac{\beta A}{d(d+\alpha +\mu_{0})}<1$, and find that there is an unstable limit cycle near the *E*_2_ when *d* = 1.483783. From the above stimulation, we know that Therorem 0.4 is not ture when $-2\sqrt{a}<c<0$.

According to an early SIR model with nonmonotone incidence rate in the literature [19], the dynamics of the system are completely determined by $R_{0}$, which means that the disease will be eliminated if $R_{0}<1$, otherwise the disease persist. Contrasting to their work and the other results for classic epidemic models, we find that the nonlinear recovery rate is also an important factor which leads to very complicated dynamics. Moreover, we find that $R_{0}$ is not enough to determine the dynamic behavior in system (5). By simulations, we predict that there would exist *b*_1*c*_ in system (3)? which has the same role as *b*_*c*_. Hopefully we can explore more relationships between the intervention actions, hospitalization conditions and spread of diseases, to provide the guidelines for public and desicion makers.
